# Sheltering Behavior and Locomotor Activity in 11 Genetically Diverse Common Inbred Mouse Strains Using Home-Cage Monitoring

**DOI:** 10.1371/journal.pone.0108563

**Published:** 2014-09-29

**Authors:** Maarten Loos, Bastijn Koopmans, Emmeke Aarts, Gregoire Maroteaux, Sophie van der Sluis, Neuro-BSIK Mouse Phenomics Consortium, Matthijs Verhage, August B. Smit

**Affiliations:** 1 Sylics (Synaptologics BV), Amsterdam, The Netherlands; 2 Department of Molecular and Cellular Neurobiology, Center for Neurogenomics and Cognitive Research (CNCR), Neuroscience Campus Amsterdam, VU University Amsterdam, Amsterdam, The Netherlands; 3 Department of Functional Genomics, Center for Neurogenomics and Cognitive Research (CNCR), Neuroscience Campus Amsterdam, VU University Amsterdam, Amsterdam, The Netherlands; 4 Section Complex Trait Genetics, Department of Clinical Genetics, VU Medical Center, Amsterdam, The Netherlands; 5 Department of Clinical Genetics, VU Medical Center, Amsterdam, The Netherlands; IGBMC/ICS, France

## Abstract

Functional genetic analyses in mice rely on efficient and in-depth characterization of the behavioral spectrum. Automated home-cage observation can provide a systematic and efficient screening method to detect unexplored, novel behavioral phenotypes. Here, we analyzed high-throughput automated home-cage data using existing and novel concepts, to detect a plethora of genetic differences in spontaneous behavior in a panel of commonly used inbred strains (129S1/SvImJ, A/J, C3H/HeJ, C57BL/6J, BALB/cJ, DBA/2J, NOD/LtJ, FVB/NJ, WSB/EiJ, PWK/PhJ and CAST/EiJ). Continuous video-tracking observations of sheltering behavior and locomotor activity were segmented into distinguishable behavioral elements, and studied at different time scales, yielding a set of 115 behavioral parameters of which 105 showed highly significant strain differences. This set of 115 parameters was highly dimensional; principal component analysis identified 26 orthogonal components with eigenvalues above one. Especially novel parameters of sheltering behavior and parameters describing aspects of motion of the mouse in the home-cage showed high genetic effect sizes. Multi-day habituation curves and patterns of behavior surrounding dark/light phase transitions showed striking strain differences, albeit with lower genetic effect sizes. This spontaneous home-cage behavior study demonstrates high dimensionality, with a strong genetic contribution to specific sets of behavioral measures. Importantly, spontaneous home-cage behavior analysis detects genetic effects that cannot be studied in conventional behavioral tests, showing that the inclusion of a few days of undisturbed, labor extensive home-cage assessment may greatly aid gene function analyses and drug target discovery.

## Introduction

Mutant mouse models are highly necessary and instrumental to reveal how human disease genes may cause aberrant behavioral phenotypes. Traditionally, mutant mice are characterized using batteries of standard behavioral tests, which when used together, can measure a large part of the behavioral spectrum, and yield substantial insight in the effect of genetic mutation. However, each of the tests introduces human interference, which largely precludes the assessment of spontaneous behavior. Yet, spontaneous home-cage activity reflects the interplay between multiple neurobiological processes (e.g. energy balance, arousal, habituation, sleep/wake cycles), each influenced by different genetic factors. Hence, assessment of spontaneous home-cage behavior has been proposed as efficient method to detect novel behavioral phenotypes in mutant mouse models [1].

Here we aimed to determine those aspects of spontaneous mouse home-cage behavior that are most influenced by genetic variation by systematically analyzing the behavior of 11 common inbred strains of mice, together covering an estimated 75% to 90% of the allelic diversity existing in the *Mus musculus* genome [2]. By comparing the between and within-strain variation in each of the home-cage behaviors of these mice, we aimed to quantify to what extent home-cage behavioral phenotypes are influenced by genetic variation. The behavior of mice was recorded for three consecutive days in a home-cage system by 24/7 overhead video tracking (PhenoTyper), in which mice are housed individually to allow for the analysis of the behavior of individual mice, essential for the assessment of within-strain variation.

In our analyses, we integrated existing methodologies with novel analyses of home-cage behavior, to scavenge a large part of the spontaneous behavioral repertoire of mice. Several measures of home-cage behavior have thus far proven useful for the detection of genetic differences between inbred strains (e.g. [3]–[7]) relevant to human disorders [8], [9], gene perturbations in knock-out lines (e.g. [10]–[12]), and genetic mouse models of human diseases, such as RETT syndrome [13], Huntington's disease [14], Spinocerebellar Ataxia type 17 [15], Down Syndrome [16] and Alzheimer's disease [17]. Although these studies used different methods for detecting behavior, predominantly video analyses or response-detectors, they commonly measured the activity of mice longitudinally, and each identified genetic effects at particular time scales. Appreciating these longitudinal genetic effects, and following the expectation that home-cage behavior is under control of multiple physiological systems acting at different time-scales, we systematically analyzed behaviors at four different time scales, ranging from multiple days to sub-minute time scales, to explore at which of these time scales genetic effects are detectable. In addition, we adopted the concept of segmentation of continuous behavioral observations (for review see [18]) to dissect activity into moves and arrests, which has not been implemented in home-cage analyses before. Finally, given that mice spend the majority of their time in a shelter/nest location in their home cage, we expanded this segmentation analysis to include sheltering behavior.

Integrating existing and novel methods to analyze home-cage behavior, the resulting data set on spontaneous behavior of 11 inbred strains of mice clearly indicated that home-cage behavior is a highly dimensional, with a strong genetic contribution to behavioral measures acting at particular time scales. Thus, provided that spontaneous home-cage behavior is studied at a sufficient level of detail, home-cage behavioral testing has the potential to detect behavioral consequences of subtle genetic manipulation, not addressed in conventional test batteries.

## Materials and Methods

### Mice

Mice were obtained from Jackson Laboratory and bred in the facilities of the NeuroBsik consortium (VU University Amsterdam, The Netherlands or Harlan Laboratories, Horst, The Netherlands; 129S1/SvImJ n = 61, A/J n = 49, BALB/cJ n = 47, C3H/HeJ n = 29, C57BL/6J n = 112, DBA/2J n = 40, FVB/NJ n = 49, NOD/ShiLtJ n = 46) or subjected to experiments 2 weeks after shipment from Jackson laboratories to the testing facility (WSB/EiJ n = 14, PWK/PhJ n = 15 and CAST/EiJ n = 14). Male 8 to 12 week old mice were singly housed on sawdust in standard Makrolon type II cages enriched with cardboard nesting material for at least one week prior to experiments, with water and food ad libitum (7:00/19:00 lights on/off; providing an abrupt phase transition). We only used male mice to avoid possible impact of estrous cycle on longitudinal behavioral assessments. Experiments were carried out in accordance with the European Communities Council Directive of 24 November 1986 (86/609/EEC), and with approval of the local animal care and use committee of the VU University Amsterdam, The Netherlands.

### Automated home-cage observation and data analyses

**Figure 1:**
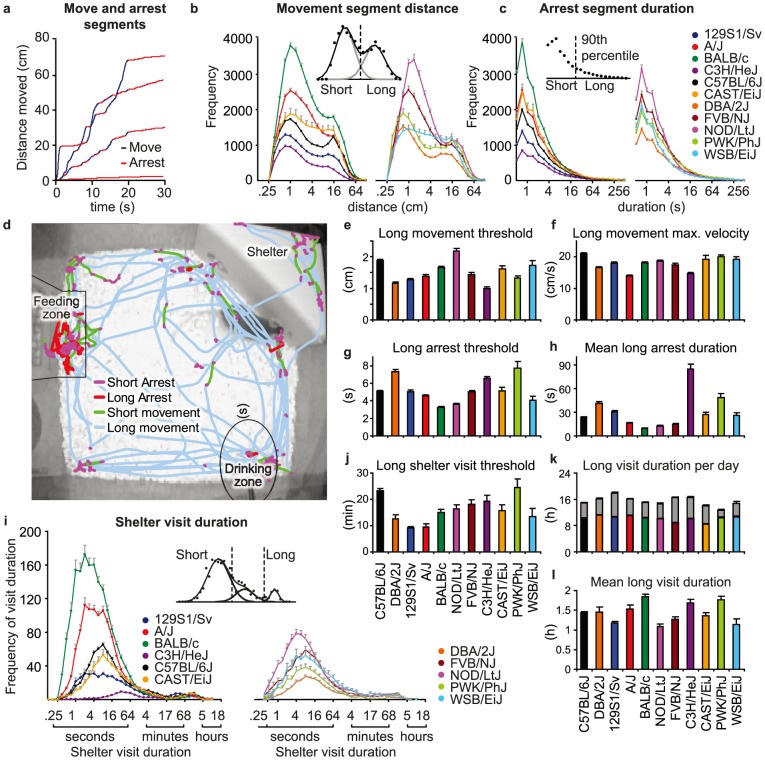
Segmentation of move, arrest and sheltering behavior. **a**) The distance moved by the center of gravity of a mouse during four 20 s segments is plotted, exemplifying arrest (red) and move (blue) segments. **b**) The length (log_2_ transformed) of all move segments during 3 days was calculated for each individual mouse, and strain averages were plotted as a histogram. The inset shows how, for a representative C57BL/6J mouse, 2 different classes of moving segments could be dissociated by the intersection of 2 Gaussians that were fitted to the distance distribution of moving segments. **c**) The duration (log_2_ transformed) of all arrest segments durations during 3 days was calculated for each individual mouse, and strain averages were plotted as a histogram. The inset shows how, for a representative C57BL/6J mouse, brief and long arrest segments were defined by an arbitrary threshold at the 90^th^ percentile of arrest durations. **d**) Visualizing a segmented track shows that short movements and long arrest occur in feeding and drinking zones. Brief arrests occur in between two long move segments, as well as in between short movements. Significant strain differences were observed in move and arrest measures, such as the **e**) long movement threshold **f**) long movement maximum velocity **g**) long arrest threshold and **h**) mean long arrest duration during the third dark phase. **i**) The duration (log_2_ transformed) of each shelter visit during 3 days was recorded for each individual mouse and strain averages were plotted as a histogram. The inset shows how, for a representative C57BL/6J mouse, the shelter visit distribution can be approximated by fitting 3 Gaussian curves. The intercept between the 2^nd^ and 3^rd^ Gaussian was taken as individually determined threshold to recognize long shelter visits. To separate brief shelter visits from other shelter visit classes a cut off duration was defined at the 90^th^ percentile of the first fitted Gaussian. Strain differences were detected in sheltering measures, such as the **j**) long (left axis) and short (right axis) shelter visit threshold, **k**) cumulative long shelter visit duration per 24-hour (grey part of bars represents dark phase) and **l**) the mean long visit duration.

Observation was performed in a home-cage environment (PhenoTyper model 3000, Noldus Information Technology, Wageningen, The Netherlands), described in detail previously [19]. The first three days in a novel PhenoTyper cage were used to analyze spontaneous behavior. Mice were introduced in the cage in the second half of the subjective light phase (14:00 h–16:00 h), and video tracking started at the onset of the first subjective dark phase (19:00 h). The cages (L = 30×W = 30×H = 35 cm) were made of transparent Perspex walls with an opaque Perspex floor covered with bedding based on cellulose. A feeding station and a water bottle were attached on to two adjacent walls. A triangular shaped shelter compartment (height: 10 cm; non-transparent material) with two entrances was fixed in the corner of the opposite two walls. The top unit of each cage contained an array of infrared LEDs and an infrared-sensitive video camera used for video-tracking. The X-Y coordinates of the center of gravity of mice, sampled at a resolution of 15 coordinates per second were acquired and smoothed using EthoVision software (EthoVision HTP 2.1.2.0, based on EthoVision XT 4.1, Noldus Information Technology, Wageningen, The Netherlands) and processed to generate behavioral parameters using AHCODA analysis software (Synaptologics BV, Amsterdam, The Netherlands). Move and arrest segments were separated by repeated running medians smoothing of X-Y coordinates (for details see [20]), using four consecutive moving windows (h1–h4) with size half-window size of h_1_ = 3, h_2_ = 2, h_3_ = h_4_ = 1, minimal size of an arrest of 3 coordinates and ε = 0.1. Smoothing settings were chosen such that move segments represented gross movements of the center of gravity, for instance, as observed during locomotor activity, turning, and rearing against the wall. Arrest segments reflected complete inactivity or minute movements of the center of gravity, for instance, caused by grooming or eating. The start of a shelter segment was recorded if the center of gravity of a mouse disappeared in the 2 cm zone drawn immediately in front of the shelter entrance. While a mouse entered or exited the shelter, the tractable portion of the mouse was rapidly iterating between being just above or below the detection threshold, and multiple entrances and exists might be registered during one entrance or exit. To counteract this, a shelter segment was only ended if the center of gravity was detected continuously for at least 7 samples (0.5 s). Three additional zones were digitally defined (see Fig. 1a); a Feeding zone around the feeding station, a Spout zone around the spout of the bottle and an OnShelter zone on top of the shelter. Previous experiments had indicated that a proportion of mice prefer to rest/sleep in a nest outside the shelter, interfering with the calculation of move and arrest parameters and time spent in specific zones. Therefore, mice which spent little time in the shelter (<60% of time in the shelter during light phase of day 2 and 3) in combination with being highly inactive outside the shelter (cumulative movement less than 2 cm per 5 min for >25% of time outside during light phase of day 2 and 3) were classified as sleeping outside the shelter and excluded from the analyses.

#### Elements of behavior identified by mouse-determined thresholds

Observation of video footage indicated that mice made short movements, such as turning or rearing against the wall, as well as long movements when mice travel from one location in the cage to the next. Observation of video footage by experimenters indicated that mice frequently visit the shelter for a few seconds (i.e., passing through) during bouts of activity. In contrast, long shelter visits during which mice appeared to be resting or sleeping, were in the range of hours. Shelter visits with intermediate durations were identified as third category. To improve the detection of spontaneous behaviors in the home-cage, we adapted existing analysis methods to segment continuous behavioral observations into distinguishable behavioral elements (for review see [18]). The goal of this analysis is to delineate separate classes of events in frequency distribution histograms of move distances (cm) and shelter segments durations (i.e. in seconds) of individual mice. Hereto, Gaussian mixture model fitting was performed to identify these separate classes, using the Solver function of Excel 2010 (Microsoft Corporation), setup to minimize the sum of squares between observed data and the mixture model (bin size of 0.5 log_2_ distance or duration). Additional information from visual inspection was implemented as limits in the Solver function, both for the move and shelter segment histograms. Visual inspection indicated that the mean of the two move-segment components were to be expected between the log_2_ distance (in cm) of −6–1 (short moves) and 1–6 (long moves). The two first shelter segment components were typically close together, to be expected between the log_2_ duration (s) of 0–10 and 9–10, whereas the third shelter component was to be expected between log_2_ duration of 10–15 (long visits).

#### Analysis of behavior at four different time-scales

Table S1 provides a detailed description of the total set of 115 parameters that were defined, including their calculation. First, activity bouts were defined, which start with a long movement and stop when a long arrest segment was encountered, or a shelter visit exceeded the brief shelter visit threshold. Characteristics of activity bouts were binned in 12 h time bins, and cumulative and mean duration and/or frequencies were calculated. Second, a habituation index for a given parameter was calculated by taking the ratio of a 12 h time bin on day 3 over day 1. Third, a DarkLight index was calculated for each parameter from the 12 h time bin values on the third day: (dark value/(dark value+light value)). Fourth, activity patterns were analyzed in terms of the change in the proportion of time active in the hours preceding and following the shift in light phase. The last and first 10 minutes of each dark and light phase were not included in parameters, to ensure that a potential asynchrony of the data streams and light regime in the testing facility would not affect these parameters.

### Statistical analyses

Before statistical analyses, parameters were log_10_ transformed in case this decreased the positive skew of the distribution (see Table S1 for parameters and the applied transformation). For each parameter, outlier data points were defined by 5 times the standard deviation of the entire dataset (i.e. not per strain), and outlying values were replaced by the 5× standard deviation upward or downward limit (a method known as Winsorizing). Estimates of the genetic effect size (narrow sense heritability) were calculated as described by Hegmann and Possidente [21] using a custom function (Microsoft Excel) as reported previously [22], which takes the differences in the number of animals per group into account when estimating the within and between-strain variance [23]. Strain differences were statistically tested using analysis of variance (ANOVA), followed by stringent Bonferroni correction for multiple testing (i.e. significance threshold of 0.05/115). In principal component (PC) analysis, principal components were retained if the Eigenvalue was larger than 1, which is typically used as threshold (Kaiser's criterion [24]). ANOVA and PCA were performed with SPSS version 20.0 (IBM, Armonk, New York, USA).

## Results

After introduction into a home-cage, novel to the mouse, spontaneous behavior of individually housed mice was continuously video-tracked at high resolution for three consecutive days. Below, the segmentation of continuous observations into elements of behavior (kinematics and sheltering) is described, followed by the analysis of behavior at four different time scales. The results of these analyses are exemplified by 20 key parameters of spontaneous behavior, which are part a larger set of 115 parameters. Of the tested 476 mice, 37 mice slept in a nest outside the shelter, as detected by experimenters as well as by our outside sleeper algorithm (see M&M and Table S2). These mice were not included in the analyses because move and arrest parameters and time spent in specific zones were severely confounded by outside sleeping. The remaining dataset contained 50,485 values (i.e. 439 mice×115 parameters) of which only 47 values (0.1%) were Winsorized (see M&M), avoiding a potential effect of this outlier handling on the strain differences described below. The data is publically available through the Mouse Phenome Database (phenome.jax.org; project data set Loos2) as well as our database (public.sylics.com; Loos2014).”

### Elements of behavior identified by mouse-determined thresholds

#### Kinematics

Whenever mice exited their shelter, video tracking produced a continuous flow of data (i.e. 15 X-Y coordinates per second). To dissect this motion data into behavioral elements, first repeated running medians smoothing of X-Y coordinates was used to identify move and arrest segments (Fig. 1a). These were further dissected, either using thresholds determined for each mouse individually (see [18]), or based on arbitrary thresholds. The frequency distribution of all move segment distances over the three days in the cage (Fig. 1b) yielded a bimodal distribution for all strains, indicating that two distinct classes of movement exist for all strains. Because these two classes were clearly distinct, we analyzed them separately. To assess these classes, Gaussian mixture model fitting of data of each individual mouse (for an example, see inset Fig. 1b) was used to define the distance threshold separating short- and long-move segments. With respect to the frequency distribution of arrest segments, no clear underlying distribution could be distinguished, and hence an arbitrary threshold was set to separate the 90% shortest arrests (short) from the 10% longest arrests (long) (Fig. 1c). A representative, dissected track of approximately 17 minutes of a C57BL/6J mouse, obtained during the dark phase of the first night in the cage (7:34:22–7:52:00) is shown in Fig. 1d. Substantial strain differences were observed in individually determined thresholds separating short and long movements (long movement threshold; Fig. 1e) and separating short and long arrests (long arrest threshold; Fig. 1g). Parameters derived of this segmentation of motion describe particular kinematic properties of mice, for example the maximum velocity of long move segments (Fig. 1f) and the mean duration of long arrests during the light phase (Fig. 1h).

#### Sheltering

With respect to sheltering behavior, long shelter visits appeared as a separate class of events in the frequency distribution of shelter visit durations (Fig. 1i). These separate classes were readily identified by Gaussian mixture model fitting (see inset in Fig. 1i). The 90th percentile of the first fitted Gaussian was used as upper threshold to distinguish short from intermediate shelter visits. Both long and short shelter visit thresholds showed substantial strain differences (Fig. 1j). Parameters that were derived of the segmentation of shelter visits describe particular aspects of sheltering behavior, for example the total time spanning long shelter visits (Fig. 1k) and the long shelter visit fraction of total shelter visits (Fig. 1l).

In conclusion, genetic differences exist between strains that affect arrests, moves and shelter visits.

### Strain differences observed on four different timescales

Next, considering that different physiological processes may act at different time scales, activity was studied at 4 time scales.

#### Activity bouts

**Figure 2:**
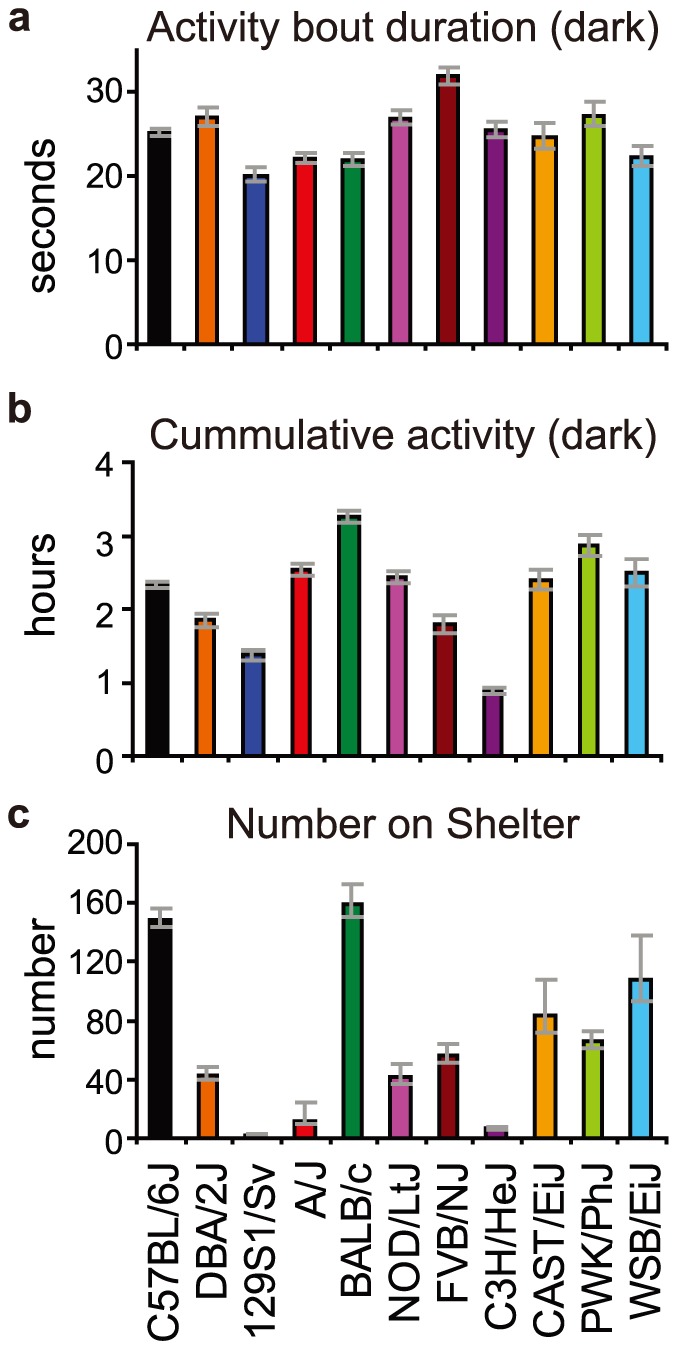
Activity bouts. Strain differences were observed in activity bout characteristics, such as **a**) the mean duration of an activity bout during the dark phase and **b**) the number of activity bouts during the dark phase and **c**) the number of jumps onto the shelter.

The duration of typical activity bouts was in the sub-minute range, and the mean duration and cumulative duration of activity bouts differed substantially among strains (Fig. 2a–b). The number of jumps on top of the shelter, which are part of an activity bout, showed a striking pattern of strain differences, with extremely low frequencies in 129S1/Sv, A/J and C3H/HeJ mice (Fig. 2c).

#### Habituation

**Figure 3:**
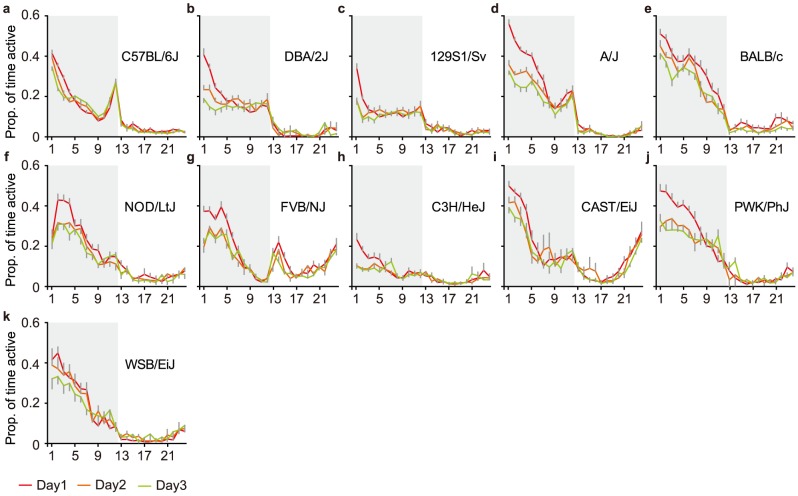
Strain-specific patterns of home-cage activity. During the three days in the home-cage environment, the strains showed different patterns with respect to multiday habituation (different line colors representing different days), differences between the proportions of activity during the dark (grey background) versus light (white background) phase, changes in activity during the hours surrounding the dark/light phase transitions.

**Figure 4:**
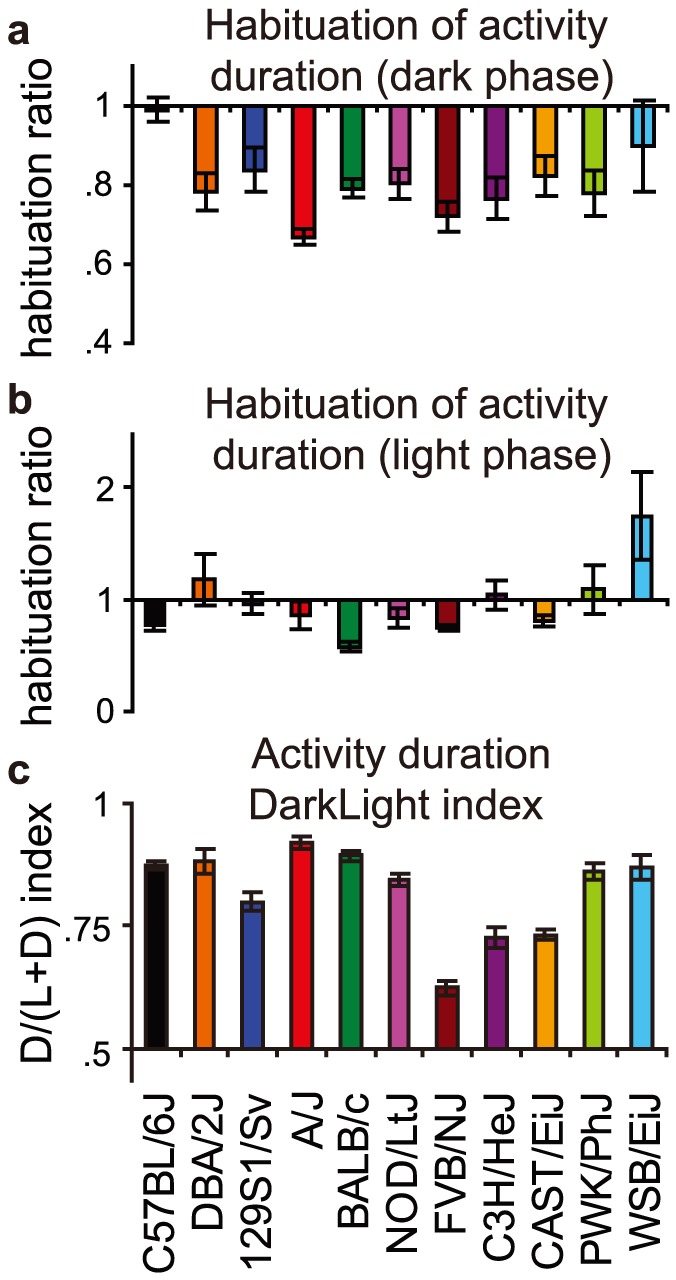
Habituation during the first three days and the effect of Light/Dark phase. **a–b**) The habituation effect across three days, in terms of the fold change from day 1 towards day 3 is plotted for dark (a) and light (b) phase. **c**) All strains have an activity duration DarkLight index above 0.5, i.e. representing more activity during the dark phase, however, substantial strain differences in this index were found.

Secondly, to evaluate habituation effects across the first three days (Fig. 3a–k), we analyzed the change in activity by taking the ratio of day 3 over day 1 (habituation index). Novelty during the first days in the PhenoTyper had strong, strain-specific effects on activity both during the dark (Fig. 4a) and light phase (Fig. 4b).

#### DarkLight index

Third, the light/dark cycle had a strong impact on behavior during each day of the experiment (see Fig. 3a–k). To assess whether a given behavior is more prominent during the dark or light phase, we defined a DarkLight index (dark value/(dark value+light value)). Substantial strain differences were detected in the DarkLight index of activity (Fig. 4c).

#### Light/dark phase transition

**Figure 5:**
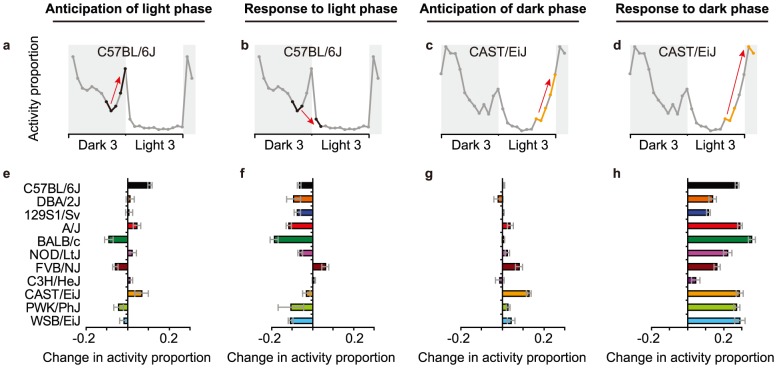
Anticipation of – and response to – phase transitions. Activity patterns were analyzed in terms of the change (slope) in the proportion of time active (activity proportion) in the few hours preceding and following the shift in light phase, and defined as the **a**) anticipation of the light phase, **b**) response to the start of the light phase, **c**) anticipation of the dark phase, **d**) response to the start of the dark phase. Each of these four slopes (i.e. change in activity proportion) showed significant strain differences (**e–h**).

Fourth, the activity of mice showed prominent changes during periods surrounding light/dark phase transition (see Fig. 3a–k). To capture strain-specific circadian patterns, we studied the behavior of mice by quantifying the anticipation and response to the onset of both light and dark phases (Fig. 5a–d). For instance, whereas C57BL/6J mice showed a sharp peak in activity towards the end of the dark phase in anticipation of the light phase, FVB/NJ mice did not to show that peak, and instead showed an increase in activity towards the end of the light phase in anticipation of the dark phase (Fig. 5e). In response to the start of the light phase, most strains significantly decreased activity, except for FVB/NJ and C3H/HeJ (Fig. 5f). Several strains showed anticipation of the dark phase, which was most pronounced in CAST/EiJ mice (Fig. 5g). Finally, all strains responded in a similar direction to the onset of the dark phase, although the magnitude of this response varied between strains (Fig. 5h).

Taken together, the analysis of behavior at multiple timescales increased the depth of phenotyping, allowing a study of strain differences across many behavioral parameters.

### Strain comparison of between-strain variation in home-cage behavior

Besides the specific 20 key parameters that were used to exemplify the segmentation of behavior into elements and longitudinal analyses mentioned above, 95 other parameters were derived of these analyses (see Table S1 and S3). Given that many of these 115 parameters are not independent (related in time or location in the cage), we applied PC analysis to reveal the methodological interdependency of the parameters. PC analysis on the data of all mice (n = 439) in the experiment identified 26 independent dimensions underlying these parameters. Thus, despite the methodological interdependence of several parameters, the identification of 26 PCs argues for the multidimensionality of the set of 115 parameters. The 20 key parameters loaded onto 10 of these 26 PCs (with loadings of 0.4 or higher), showing that the entire set of 115 parameters certainly covers more variation than contained in the set of 20 parameters used to exemplify the analyses (varimax rotated PC solution; Table S4).

**Figure 6:**
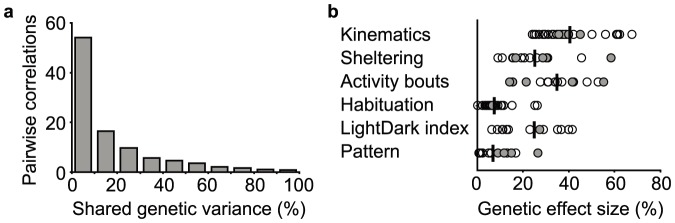
Genetic influences on spontaneous home-cage behavior. **a**) A histogram of the number of pairwise correlations among 115 parameters, with a particular shared genetic variance. **b**) The genetic effect size for each of the 115 parameter (bullets, with 20 key parameters grey filled) was calculated, and grouped per behavioral domain. The vertical mark represents the average genetic effect size of a category.

Next, we investigated to what extent these parameters detected common or unique genetic variation. Differences between inbred mouse strains result from genetic effects, thus, besides methodological interdependency of parameters as described above, high correlation between parameters across strain means would indicate that these measures are controlled by common genetic effects. This was quantified by square of the Pearson correlation (r^2^). The percentage of genetic variance shared between any two parameters of the set of 115 parameters is plotted as a frequency distribution in Figure 6a. The majority of pairwise correlations (>90%) have a percentage of shared genetic variance less than 50%. Thus, even despite contribution of methodological interdependency of parameters, the majority of 115 parameters detect genetic variance that is not substantially covered by another parameter.

Of the total set of 115 parameters, 105 showed highly significant Bonferroni corrected (P<4.35*10^−4^) strain differences (including all 20 key parameters), with genetic effect sizes ranged from 24% to 67% (Table S3), indicating substantial genetic contribution to spontaneous behavioral phenotypes. There was an overall difference in the genetic effect size when parameters were grouped according to their respective categories (Fig. 6b; F(5,109) = 35.4, p<0.001). The two categories of parameters reflecting the segmentation of behavior into elements showed the higher average genetic effect size (Fig. 6b). Parameters describing habituation effects and anticipation of – and response to – light/dark phase transitions showed the lowest genetic effect size.

## Discussion

The integrated analyses of novel and existing analyses of spontaneous behavior of individually housed mice in their home-cage provided a comprehensive overview of spontaneous behavioral phenotypes of 11 commonly used inbred strains of mice. A set of 20 parameters was used to exemplify the analysis of the behavioral repertoire of mice in their home-cage. Spontaneous home-cage behavior was highly dimensional, with a strong genetic contribution to particular sets of behavioral measures acting at particular time scales.

We segmented continuous, 3-day video-tracking data of spontaneous behaviors in the home-cage into distinguishable elements. Besides segmenting movement into kinematic parameters using individually customized cutoff points (for review see [18]), we for the first time applied this analysis to sheltering behavior of mice. These thresholds, used to separate different types of move, arrest and shelter segments, were highly instrumental to establish significant strain differences. Parameters describing kinematics, and the novel parameters of sheltering behavior and the activity bout characteristics directly derived from these thresholds showed high genetic effect sizes, showing these groups of parameters are sensitive to detect genetic differences between strains. Therefore, future studies aimed at detecting the effect of genetic perturbations using video-tracking systems may strongly benefit from the inclusion of similar kinematic and sheltering analyses.

Changes in behavior as a consequence of habituation were observed that progressed during the first 3 days in the cage. These findings are in line with habituation periods in previous home-cage observations [3], [25], as well as habituation of at least a few hours in a recent study with a large open arena [26]. In contrast to the multi-day habituation profiles, in conventional assays, such as an open field, habituation is typically described in terms of a reduction in activity at a time scale of minutes after introduction into the apparatus. Thus, short-lived conventional assays only probe the very initial stage of a habituation process, which, as can be concluded from our data, take in total up to three days depending on the mouse strain used.

Within each day there is a major influence of light/dark rhythm on most measures, as determined in the present study by the DarkLight index. The activity of A/J mice was most influenced by the light regime, with 92% of its activity occurring during the dark phase. In conventional anxiety assays under illuminated conditions, such as open field, A/J mice are often described as one of the most inactive strains (e.g. [27]–[31]), suggesting that high absolute values on the activity DarkLight index may predict hypo activity in conventional anxiety assays. In contrast to A/J, the strains FVB/NJ, C3H/HeJ and CAST/EiJ had lower values on the DarkLight indices, which might relate to the poor vision of these strains.

The activity of mice changed during periods around phase transition. Besides strain differences in particular aspects of moving and sheltering behavior, there were strain-specific patterns of activity during periods around light/dark phase transition. One of the most striking phenotypes of C57BL/6J mice is their increase in activity towards the end of the dark phase, which is not observed for other strains in the current study, but found for CAST/EiJ and MSM/Ms mice in a previous study [32]. Low metabolic status at the end of the dark phase in C57BL/6J and CAST/EiJ mice, which might promote food intake behavior and concomitant activity, was probably not underlying this anticipation because the proportion of time spent in the feeding zone did not increase (supplemental info). During light/dark phase transitions, it is likely that numerous physiological processes interact to prepare for, and respond to, the major change in behavioral output required for dark and light phases. Each of these physiological processes is influenced by genetic variants, which could explain the different and complex pattern between strains during periods around light/dark phase transition.

Although significant strain differences were detected in behavioral changes across multiple days (habituation group) and in anticipation of – and response to – light/dark phase transitions (pattern group), the genetic effect sizes of these parameters were generally lower. Apparently, the vast amount of genetic variation between the strains used in the current study did not affect these aspects of behavior to large extent.

The OnShelter visit parameter described a highly significant difference in behavior; some strains almost never climbed on top of the shelter (i.e. 129S1/Sv, A/J and C3H/HeJ). Given that these strains do not appear to have poorer motor performance on an accelerating rotarod or less grip strength compared to for instance DBA/2J mice [33], [34], impaired motor function is not a probable explanation for these phenotypic differences among strains.

We chose to first focus on describing the unique behavioral profiles of commonly used inbred strains of mice, and by using extensive data analysis, measure the richness of their behavioral repertoire obtained in the home-cage. We feel that it is timely and necessary to demonstrate that home-cage measurements can reliably detect strain differences and probe different aspects of behavior, which should be a firm basis for subsequent experiments. The behavioral analysis was carried out in a particular home-cage setting, which may render the obtained patterns of strain differences home-cage specific. In addition, the currently limited possibility to video-track and distinguish socially house mice required us to house mice individually, impacting on mouse behavior and strain differences. Nonetheless, it is anticipated that studies, ranging from gene perturbations, brain lesions and pharmacological interventions, using the methodology described will provide biological underpinning of particular parameters as measured here and their relevance in the context of human diseases.

In conclusion, thorough analysis of spontaneous home-cage behavior detects numerous genetic effects that are not studied in conventional behavioral tests targeted at a particular behavioral domain. We envision that the inclusion of a few days of undisturbed, labor extensive home-cage assessment in behavioral screening programs will greatly increase the discriminative power of such programs, and will aid gene function analyses and drug target discovery.

## Supporting Information

Table S1 **Detailed description per measure.**
(PDF)

Table S2 **Number of outside sleepers per strain.**
(PDF)

Table S3 **Test statistics per measure.**
(PDF)

Table S4 **PC analysis varimax rotated solution matrix. 20 key parameters are indicated (bold).**
(PDF)
