# Supplementary material for: Sheltering Behavior and Locomotor Activity in 11 Genetically Diverse Common Inbred Mouse Strains Using Home-Cage Monitoring
Source: PLoS One. 2014 Sep 29;9(9):e108563. doi: 10.1371/journal.pone.0108563 (PMC4180925; doi:10.1371/journal.pone.0108563)
Supplement: Table S1 — Detailed description per measure. (PDF) [file pone.0108563.s001.pdf]

Segmentation of behavior

Nr	Abreviated name in Manuscript (and on website)	Segment	Transformation	Units	Calculation	Limits	Explanation
1	Long movement threshold	Move	Log10	cm		none	Cut-off value to separate short and long movements
2	Long movement max. velocity	Move	Log10	cm/s		none	Average velocity of the 95th percentile fastest long movement segments
3	Long movement fraction of total movement	Move	None	fraction		none	The fraction of movement segments with distance larger than long movement threshold
4	Long movement distance - dark	Move	Log10	cm		none	Cummulative long movement distance during the dark phase
5	Long movement distance - light	Move	Log10	cm		none	Cummulative long movement distance during the light phase
6	Long movement number - dark	Move	Log10	frequency		none	Cummulative long movement number during the dark phase
7	Long movement number - light	Move	Log10	frequency		none	Cummulative long movement number during the light phase
8	Mean long movement distance - dark	Move	Log10	cm		Number of occurances during dark phase 3 > 10	Mean distance per long movement during the dark phase
9	Mean long movement distance - light	Move	Log10	cm		Number of occurances during light phase 3 > 10	Mean distance per long movement during the light phase
10	Mean short movement distance - dark	Move	Log10	cm		Number of occurances during dark phase 3 > 10	Mean distance per short movement during the dark phase
11	Mean short movement distance - light	Move	Log10	cm		Number of occurances during light phase 3 > 10	Mean distance per short movement during the light phase
12	Short movement distance - dark	Move	Log10	cm		none	Cummulative short movement distance during the dark phase
13	Short movement distance - light	Move	Log10	cm		none	Cummulative short movement distance during the light phase
14	Short movement number - dark	Move	Log10	frequency		none	Cummulative short movement number during the dark phase
15	Short movement number - light	Move	Log10	frequency		none	Cummulative short movement number during the light phase
16	Long arrest threshold	Arrest	Log10	s		none	Cut-off value to separate short and long arrests
17	Long arrest duration - dark	Arrest	Log10	s		none	Cummulative duration of long arrests during the dark phase
18	Long arrest duration - light	Arrest	None	s		none	Cummulative duration of long arrests during the light phase
19	Long arrest number - dark	Arrest	Log10	frequency		none	Cummulative number of long arrests during the dark phase
20	Long arrest number - light	Arrest	Log10	frequency		none	Cummulative number of long arrests during the light phase
21	Mean long arrest duration - dark	Arrest	Log10	s		Number of occurances during dark phase 3 > 10	Mean duration per long arrest during the dark phase
22	Mean long arrest duration - light	Arrest	Log10	s		Number of occurances during light phase 3 > 10	Mean duration per long arrest during the light phase
23	Mean short arrest duration - dark	Arrest	Log10	s		Number of occurances during dark phase 3 > 10	Mean duration per short arrest during the dark phase
24	Mean short arrest duration - light	Arrest	Log10	s		Number of occurances during light phase 3 > 10	Mean duration per short arrest during the light phase
25	Short arrest duration - dark	Arrest	None	s		none	Cummulative duration of short arrests during the dark phase
26	Short arrest duration - light	Arrest	Log10	s		none	Cummulative duration of short arrests during the light phase
27	Short arrest number - dark	Arrest	Log10	frequency		none	Cummulative number of short arrests during the dark phase
28	Short arrest number - light	Arrest	Log10	frequency		none	Cummulative number of short arrests during the light phase
29	Long shelter visit threshold	Sheltering	None	s		none	Cut-off value to separate intermediate and long shelter visits
30	Short shelter visit threshold	Sheltering	None	s		none	Cut-off value to separate short and intermediate shelter visits
31	Long shelter visit duration - dark	Sheltering	None	s		none	Cummulative duration of long shelter visits during the dark phase
32	Long shelter visit duration - light	Sheltering	None	s		none	Cummulative duration of long shelter visits during the light phase
33	Long shelter visit fraction of total visits	Sheltering	Log10	fraction		none	The fraction of shelter visits with duration longer than long shelter visit threshold
34	Long shelter visit number - dark	Sheltering	Log10	frequency		none	Cummulative number of long shelter visits during the dark phase
35	Long shelter visit number - light	Sheltering	Log10	frequency		none	Cummulative number of long shelter visits during the light phase
36	Mean long shelter visit duration	Sheltering	Log10	s		Number of long shelter visits during first 3 days > 10	Mean duration per long shelter visit during the first 3 days
37	Mean short shelter visit duration - dark	Sheltering	Log10	s		Number of occurances during dark phase 3 > 10	Mean duration per short shelter visit during the dark phase
38	Mean short shelter visit duration - light	Sheltering	Log10	s		Number of occurances during light phase 3 > 10	Mean duration per short shelter visit during the light phase
39	Short shelter visit duration - dark	Sheltering	None	s		none	Cummulative duration of short shelter visits during the dark phase
40	Short shelter visit duration - light	Sheltering	Log10	s		none	Cummulative duration of short shelter visits during the light phase
41	Short shelter visit number - dark	Sheltering	Log10	frequency		none	Cummulative number of short shelter visits during the dark phase
42	Short shelter visit number - light	Sheltering	Log10	frequency		none	Cummulative number of short shelter visits during the light phase

Temporal aspects

Nr	Abreviated name in Manuscript (and on website)	Temporal aspect	Transformation	Units	Calculation	Limits	Explanation
43	Activity duration - habituation ratio dark	Habituation	Log10	ratio	[(parameter value on day 3)/(parameter value on day 1)]	parameter value on day 1 > 60 seconds	Habituation effect: Change in cummulative activity duration during the dark phase of day 3 compared to the dark phase of day 1
44	Feeding zone duration - habituation ratio dark	Habituation	Log10	ratio	[(parameter value on day 3)/(parameter value on day 1)]	parameter value on day 1 > 60 seconds	Habituation effect: Change in cummulative Feeding zone duration during the dark phase of day 3 compared to the dark phase of day 1
45	Long shelter visit duration - habituation ratio dark	Habituation	Log10	ratio	[(parameter value on day 3)/(parameter value on day 1)]	parameter value on day 1 > 5400 seconds	Habituation effect: Change in cummulative long shelter visit duration during the dark phase of day 3 compared to the dark phase of day 1
46	OnShelter zone duration - habituation ratio dark	Habituation	Log10	ratio	[(parameter value on day 3)/(parameter value on day 1)]	parameter value on day 1 > 60 seconds	Habituation effect: Change in cummulative OnShelter zone duration during the dark phase of day 3 compared to the dark phase of day 1
47	Mean short movement distance - habituation ratio dark	Habituation	Log10	ratio	[(parameter value on day 3)/(parameter value on day 1)]	none	Habituation effect: Change in mean short movement distance during the dark phase of day 3 compared to the dark phase of day 1
48	Mean activity duration - habituation ratio dark	Habituation	Log10	ratio	[(parameter value on day 3)/(parameter value on day 1)]	none	Habituation effect: Change in mean activity duration during the dark phase of day 3 compared to the dark phase of day 1
49	Activity number - habituation ratio dark	Habituation	Log10	ratio	[(parameter value on day 3)/(parameter value on day 1)]	parameter value on day 1 > 10	Habituation effect: Change in cummulative activity number during the dark phase of day 3 compared to the dark phase of day 1
50	Mean short arrest duration - habituation ratio dark	Habituation	Log10	ratio	[(parameter value on day 3)/(parameter value on day 1)]	none	Habituation effect: Change in mean short arrest duration during the dark phase of day 3 compared to the dark phase of day 1
51	Long arrest duration - habituation ratio dark	Habituation	Log10	ratio	[(parameter value on day 3)/(parameter value on day 1)]	parameter value on day 1 > 60 seconds	Habituation effect: Change in cummulative long arrest long arrest duration during the dark phase of day 3 compared to the dark phase of day 1
52	Mean long arrest duration - habituation ratio dark	Habituation	Log10	ratio	[(parameter value on day 3)/(parameter value on day 1)]	none	Habituation effect: Change in mean long arrest duration during the dark phase of day 3 compared to the dark phase of day 1
53	Long arrest number - habituation ratio dark	Habituation	Log10	ratio	[(parameter value on day 3)/(parameter value on day 1)]	parameter value on day 1 > 10	Habituation effect: Change in cummulative long arrest long arrest number during the dark phase of day 3 compared to the dark phase of day 1
54	Mean short shelter visit duration - habituation ratio dark	Habituation	Log10	ratio	[(parameter value on day 3)/(parameter value on day 1)]	none	Habituation effect: Change in mean short shelter visit duration during the dark phase of day 3 compared to the dark phase of day 1
55	Mean long movement distance - habituation ratio dark	Habituation	Log10	ratio	[(parameter value on day 3)/(parameter value on day 1)]	none	Habituation effect: Change in mean long movement distance during the dark phase of day 3 compared to the dark phase of day 1
56	Spout zone duration - habituation ratio dark	Habituation	Log10	ratio	[(parameter value on day 3)/(parameter value on day 1)]	parameter value on day 1 > 60 seconds	Habituation effect: Change in cummulative Spout zone duration during the dark phase of day 3 compared to the dark phase of day 1
57	Activity duration - habituation ratio light	Habituation	Log10	ratio	[(parameter value on day 3)/(parameter value on day 1)]	parameter value on day 1 > 60 seconds	Habituation effect: Change in cummulative activity duration during the light phase of day 3 compared to the light phase of day 1
58	Feeding zone duration - habituation ratio light	Habituation	Log10	ratio	[(parameter value on day 3)/(parameter value on day 1)]	parameter value on day 1 > 60 seconds	Habituation effect: Change in cummulative Feeding zone duration during the light phase of day 3 compared to the light phase of day 1
59	Long shelter visit duration - habituation ratio light	Habituation	Log10	ratio	[(parameter value on day 3)/(parameter value on day 1)]	parameter value on day 1 > 5400 seconds	Habituation effect: Change in cummulative long shelter visit duration during the light phase of day 3 compared to the light phase of day 1
60	OnShelter zone duration - habituation ratio light	Habituation	Log10	ratio	[(parameter value on day 3)/(parameter value on day 1)]	parameter value on day 1 > 60 seconds	Habituation effect: Change in cummulative OnShelter zone duration during the light phase of day 3 compared to the light phase of day 1
61	Mean short movement distance - habituation ratio light	Habituation	Log10	ratio	[(parameter value on day 3)/(parameter value on day 1)]	none	Habituation effect: Change in mean short movement distance during the light phase of day 3 compared to the light phase of day 1
62	Mean activity duration - habituation ratio light	Habituation	Log10	ratio	[(parameter value on day 3)/(parameter value on day 1)]	none	Habituation effect: Change in mean activity duration during the light phase of day 3 compared to the light phase of day 1
63	Activity number - habituation ratio light	Habituation	Log10	ratio	[(parameter value on day 3)/(parameter value on day 1)]	parameter value on day 1 > 10	Habituation effect: Change in cummulative activity number during the light phase of day 3 compared to the light phase of day 1
64	Mean short arrest duration - habituation ratio light	Habituation	Log10	ratio	[(parameter value on day 3)/(parameter value on day 1)]	none	Habituation effect: Change in mean short arrest duration during the light phase of day 3 compared to the light phase of day 1
65	Long arrest duration - habituation ratio light	Habituation	Log10	ratio	[(parameter value on day 3)/(parameter value on day 1)]	parameter value on day 1 > 60 seconds	Habituation effect: Change in cummulative long arrest long arrest duration during the light phase of day 3 compared to the light phase of day 1
66	Mean long arrest duration - habituation ratio light	Habituation	Log10	ratio	[(parameter value on day 3)/(parameter value on day 1)]	none	Habituation effect: Change in mean long arrest duration during the light phase of day 3 compared to the light phase of day 1
67	Long arrest number - habituation ratio light	Habituation	Log10	ratio	[(parameter value on day 3)/(parameter value on day 1)]	parameter value on day 1 > 10	Habituation effect: Change in cummulative long arrest long arrest number during the light phase of day 3 compared to the light phase of day 1
68	Mean short shelter visit duration - habituation ratio light	Habituation	Log10	ratio	[(parameter value on day 3)/(parameter value on day 1)]	none	Habituation effect: Change in mean short shelter visit duration during the light phase of day 3 compared to the light phase of day 1
69	Mean long movement distance - habituation ratio light	Habituation	Log10	ratio	[(parameter value on day 3)/(parameter value on day 1)]	none	Habituation effect: Change in mean long movement distance during the light phase of day 3 compared to the light phase of day 1
70	Spout zone duration - habituation ratio light	Habituation	Log10	ratio	[(parameter value on day 3)/(parameter value on day 1)]	parameter value on day 1 > 60 seconds	Habituation effect: Change in cummulative Spout zone duration during the light phase of day 3 compared to the light phase of day 1
71	Activity duration - darklight index	DarkLightIndex	None	index	[(parameter value during dark phase of day 3)/((parameter value during dark phase of day 3)+(parameter value during light phase of day 3))]	[(parameter value during dark phase of day 3)+(parameter value during light phase of day 3)]>0	Effect of light regime: Index describing the difference in cummulative activity duration between light and dark phase. If this index is between 0.5 and 1 then parameter values are higher during the dark phase. If this index is between 0 and 0.5 then param
72	Long shelter visit duration - darklight index	DarkLightIndex	None	index	[(parameter value during dark phase of day 3)/((parameter value during dark phase of day 3)+(parameter value during light phase of day 3))]	[(parameter value during dark phase of day 3)+(parameter value during light phase of day 3)]>9	Effect of light regime: Index describing the difference in cummulative long shelter visit duration between light and dark phase. If this index is between 0.5 and 1 then parameter values are higher during the dark phase. If this index is between 0 and 0.5
73	Mean short movement distance - darklight index	DarkLightIndex	None	index	[(parameter value during dark phase of day 3)/((parameter value during dark phase of day 3)+(parameter value during light phase of day 3))]	[(parameter value during dark phase of day 3)+(parameter value during light phase of day 3)]>12	Effect of light regime: Index describing the difference in mean short movement distance between light and dark phase. If this index is between 0.5 and 1 then parameter values are higher during the dark phase. If this index is between 0 and 0.5 then parame
74	Feeding zone duration - darklight index	DarkLightIndex	None	index	[(parameter value during dark phase of day 3)/((parameter value during dark phase of day 3)+(parameter value during light phase of day 3))]	[(parameter value during dark phase of day 3)+(parameter value during light phase of day 3)]>7	Effect of light regime: Index describing the difference in cummulative Feeding zone duration between light and dark phase. If this index is between 0.5 and 1 then parameter values are higher during the dark phase. If this index is between 0 and 0.5 then p
75	Mean activity duration - darklight index	DarkLightIndex	None	index	[(parameter value during dark phase of day 3)/((parameter value during dark phase of day 3)+(parameter value during light phase of day 3))]	[(parameter value during dark phase of day 3)+(parameter value during light phase of day 3)]>1	Effect of light regime: Index describing the difference in mean activity duration between light and dark phase. If this index is between 0.5 and 1 then parameter values are higher during the dark phase. If this index is between 0 and 0.5 then parameter va
76	Activity number - darklight index	DarkLightIndex	None	index	[(parameter value during dark phase of day 3)/((parameter value during dark phase of day 3)+(parameter value during light phase of day 3))]	[(parameter value during dark phase of day 3)+(parameter value during light phase of day 3)]>2	Effect of light regime: Index describing the difference in cummulative activity number between light and dark phase. If this index is between 0.5 and 1 then parameter values are higher during the dark phase. If this index is between 0 and 0.5 then paramet
77	Mean short arrest duration - darklight index	DarkLightIndex	None	index	[(parameter value during dark phase of day 3)/((parameter value during dark phase of day 3)+(parameter value during light phase of day 3))]	[(parameter value during dark phase of day 3)+(parameter value during light phase of day 3)]>3	Effect of light regime: Index describing the difference in mean short arrest duration between light and dark phase. If this index is between 0.5 and 1 then parameter values are higher during the dark phase. If this index is between 0 and 0.5 then paramete
78	Long arrest duration - darklight index	DarkLightIndex	None	index	[(parameter value during dark phase of day 3)/((parameter value during dark phase of day 3)+(parameter value during light phase of day 3))]	[(parameter value during dark phase of day 3)+(parameter value during light phase of day 3)]>4	Effect of light regime: Index describing the difference in cummulative long arrest long arrest duration between light and dark phase. If this index is between 0.5 and 1 then parameter values are higher during the dark phase. If this index is between 0 and
79	Mean long arrest duration - darklight index	DarkLightIndex	None	index	[(parameter value during dark phase of day 3)/((parameter value during dark phase of day 3)+(parameter value during light phase of day 3))]	[(parameter value during dark phase of day 3)+(parameter value during light phase of day 3)]>5	Effect of light regime: Index describing the difference in mean long arrest duration between light and dark phase. If this index is between 0.5 and 1 then parameter values are higher during the dark phase. If this index is between 0 and 0.5 then parameter
80	Long arrest number - darklight index	DarkLightIndex	None	index	[(parameter value during dark phase of day 3)/((parameter value during dark phase of day 3)+(parameter value during light phase of day 3))]	[(parameter value during dark phase of day 3)+(parameter value during light phase of day 3)]>6	Effect of light regime: Index describing the difference in cummulative long arrest long arrest number between light and dark phase. If this index is between 0.5 and 1 then parameter values are higher during the dark phase. If this index is between 0 and 0
81	Mean short shelter visit duration - darklight index	DarkLightIndex	None	index	[(parameter value during dark phase of day 3)/((parameter value during dark phase of day 3)+(parameter value during light phase of day 3))]	[(parameter value during dark phase of day 3)+(parameter value during light phase of day 3)]>8	Effect of light regime: Index describing the difference in mean short shelter visit duration between light and dark phase. If this index is between 0.5 and 1 then parameter values are higher during the dark phase. If this index is between 0 and 0.5 then p
82	Long shelter visit number - darklight index	DarkLightIndex	None	index	[(parameter value during dark phase of day 3)/((parameter value during dark phase of day 3)+(parameter value during light phase of day 3))]	[(parameter value during dark phase of day 3)+(parameter value during light phase of day 3)]>10	Effect of light regime: Index describing the difference in cummulative long shelter visit number between light and dark phase. If this index is between 0.5 and 1 then parameter values are higher during the dark phase. If this index is between 0 and 0.5 th
83	Mean long movement distance - darklight index	DarkLightIndex	None	index	[(parameter value during dark phase of day 3)/((parameter value during dark phase of day 3)+(parameter value during light phase of day 3))]	[(parameter value during dark phase of day 3)+(parameter value during light phase of day 3)]>11	Effect of light regime: Index describing the difference in mean long movement distance between light and dark phase. If this index is between 0.5 and 1 then parameter values are higher during the dark phase. If this index is between 0 and 0.5 then paramet

Temporal aspects (continued)

Nr	Abreviated name in Manuscript (and on website)	Temporal aspect	Transformation	Units	Calculation	Limits	Explanation
84	OnShelter zone duration - darklight index	DarkLightIndex	None	index	[(parameter value during dark phase of day 3)/((parameter value during dark phase of day 3)+(parameter value during light phase of day 3))]	[(parameter value during dark phase of day 3)+(parameter value during light phase of day 3)]>13	Effect of light regime: Index describing the difference in cummulative OnShelter zone duration between light and dark phase. If this index is between 0.5 and 1 then parameter values are higher during the dark phase. If this index is between 0 and 0.5 then
85	Spout zone duration - darklight index	DarkLightIndex	None	index	[(parameter value during dark phase of day 3)/((parameter value during dark phase of day 3)+(parameter value during light phase of day 3))]	[(parameter value during dark phase of day 3)+(parameter value during light phase of day 3)]>14	Effect of light regime: Index describing the difference in cummulative Spout zone duration between light and dark phase. If this index is between 0.5 and 1 then parameter values are higher during the dark phase. If this index is between 0 and 0.5 then par
86	Activity change in anticipation of light	Anticipation / response to phase transition	None	fraction of total time	[(Activity duration during hours 11 and 12 of the dark phase in seconds)/(2*3600)]-[(Activity duration during hours 8, 9 and 10 of the dark phase in seconds)/(3*3600)]	(Cummulative activity during hours 8,9 and 10) > 0 and (Cummulative activity during hours 11 and 12) > 0	Change in activity during the last 5 hours of the dark phase in anticipation of the upcoming light phase
87	Activity change in response to to light	Anticipation / response to phase transition	None	fraction of total time	[(Activity duration during hours 1 and 2 of the light phase in seconds)/(2*3600)]-[(Activity duration during hours 8, 9 and 10 of the dark phase in seconds)/(3*3600)]	(Cummulative activity during hours 8,9 and 10) > 0 and (Cummulative activity during hours 11 and 12) > 0	Change in activity during the first 2 hours of the light phase in response to the onset of the light phase
88	Activity change in anticipation of dark	Anticipation / response to phase transition	None	fraction of total time	[(Activity duration during hours 11 and 12 of the light phase in seconds)/(2*3600)]-[(Activity duration during hours 8, 9 and 10 of the light phase in seconds)/(3*3600)]	(Cummulative activity during hours 8,9 and 10) > 0 and (Cummulative activity during hours 11 and 12) > 0	Change in activity during the last 5 hours of the light phase in anticipation of the upcoming dark phase
89	Activity change in response to to dark	Anticipation / response to phase transition	None	fraction of total time	[(Activity duration during hours 1 and 2 of the dark phase in seconds)/(2*3600)]-[(Activity duration during hours 8, 9 and 10 of the light phase in seconds)/(3*3600)]	(Cummulative activity during hours 8,9 and 10) > 0 and (Cummulative activity during hours 11 and 12) > 0	Change in activity during the first 2 hours of the dark phase in response to the onset of the dark phase
90	Feeding zone change in anticipation light	Anticipation / response to phase transition	None	fraction of time outside	[(Feeding zone duration during hours 1 and 2 of the dark phase in seconds)/(time outside)]-[(Feeding zone duration during hours 8, 9 and 10 of the light phase in seconds)/(time outside)]	(Cummulative Feeding zone duration during hours 8,9 and 10) > 0 and (Cummulative Feeding zone duration during hours 11 and 12) > 0	Change in Feeding zone during the first 2 hours of the dark phase in response to the onset of the dark phase
91	Feeding zone change in response to light	Anticipation / response to phase transition	None	fraction of time outside	[(Feeding zone duration during hours 1 and 2 of the light phase in seconds)/(time outside)]-[(Feeding zone duration during hours 8, 9 and 10 of the dark phase in seconds)/(time outside)]	(Cummulative Feeding zone duration during hours 8,9 and 10) > 0 and (Cummulative Feeding zone duration during hours 11 and 12) > 0	Change in Feeding zone during the first 2 hours of the light phase in response to the onset of the light phase
92	Feeding zone change in anticipation dark	Anticipation / response to phase transition	None	fraction of time outside	[(Feeding zone duration during hours 11 and 12 of the light phase in seconds)/(time outside)]-[(Feeding zone duration during hours 8, 9 and 10 of the light phase in seconds)/(time outside)]	(Cummulative Feeding zone duration during hours 8,9 and 10) > 0 and (Cummulative Feeding zone duration during hours 11 and 12) > 0	Change in Feeding zone during the last 5 hours of the light phase in anticipation of the upcoming dark phase
93	Feeding zone change in response to dark	Anticipation / response to phase transition	None	fraction of time outside	[(Feeding zone duration during hours 11 and 12 of the dark phase in seconds)/(time outside)]-[(Feeding zone duration during hours 8, 9 and 10 of the dark phase in seconds)/(time outside)]	(Cummulative Feeding zone duration during hours 8,9 and 10) > 0 and (Cummulative Feeding zone duration during hours 11 and 12) > 0	Change in Feeding zone during the last 5 hours of the dark phase in anticipation of the upcoming light phase
94	OnShelter zone change in anticipation light	Anticipation / response to phase transition	None	fraction of time outside	[(OnShelter zone duration during hours 1 and 2 of the dark phase in seconds)/(time outside)]-[(OnShelter zone duration during hours 8, 9 and 10 of the light phase in seconds)/(time outside)]	(Cummulative OnShelter zone duration during hours 8,9 and 10) > 0 and (Cummulative OnShelter zone duration during hours 11 and 12) > 0	Change in OnShelter zone during the first 2 hours of the dark phase in response to the onset of the dark phase
95	OnShelter zone change in response to light	Anticipation / response to phase transition	None	fraction of time outside	[(OnShelter zone duration during hours 1 and 2 of the light phase in seconds)/(time outside)]-[(OnShelter zone duration during hours 8, 9 and 10 of the dark phase in seconds)/(time outside)]	(Cummulative OnShelter zone duration during hours 8,9 and 10) > 0 and (Cummulative OnShelter zone duration during hours 11 and 12) > 0	Change in OnShelter zone during the first 2 hours of the light phase in response to the onset of the light phase
96	OnShelter zone change in anticipation dark	Anticipation / response to phase transition	None	fraction of time outside	[(OnShelter zone duration during hours 11 and 12 of the light phase in seconds)/(time outside)]-[(OnShelter zone duration during hours 8, 9 and 10 of the light phase in seconds)/(time outside)]	(Cummulative OnShelter zone duration during hours 8,9 and 10) > 0 and (Cummulative OnShelter zone duration during hours 11 and 12) > 0	Change in OnShelter zone during the last 5 hours of the light phase in anticipation of the upcoming dark phase
97	OnShelter zone change in response to dark	Anticipation / response to phase transition	None	fraction of time outside	[(OnShelter zone duration during hours 11 and 12 of the dark phase in seconds)/(time outside)]-[(OnShelter zone duration during hours 8, 9 and 10 of the dark phase in seconds)/(time outside)]	(Cummulative OnShelter zone duration during hours 8,9 and 10) > 0 and (Cummulative OnShelter zone duration during hours 11 and 12) > 0	Change in OnShelter zone during the last 5 hours of the dark phase in anticipation of the upcoming light phase
98	Spout zone change in anticipation light	Anticipation / response to phase transition	None	fraction of time outside	[(Spout zone duration during hours 1 and 2 of the dark phase in seconds)/(time outside)]-[(Spout zone duration during hours 8, 9 and 10 of the light phase in seconds)/(time outside)]	(Cummulative Spout zone duration during hours 8,9 and 10) > 0 and (Cummulative Spout zone duration during hours 11 and 12) > 0	Change in Spout zone during the first 2 hours of the dark phase in response to the onset of the dark phase
99	Spout zone change in response to light	Anticipation / response to phase transition	None	fraction of time outside	[(Spout zone duration during hours 1 and 2 of the light phase in seconds)/(time outside)]-[(Spout zone duration during hours 8, 9 and 10 of the dark phase in seconds)/(time outside)]	(Cummulative Spout zone duration during hours 8,9 and 10) > 0 and (Cummulative Spout zone duration during hours 11 and 12) > 0	Change in Spout zone during the first 2 hours of the light phase in response to the onset of the light phase
100	Spout zone change in anticipation dark	Anticipation / response to phase transition	None	fraction of time outside	[(Spout zone duration during hours 11 and 12 of the light phase in seconds)/(time outside)]-[(Spout zone duration during hours 8, 9 and 10 of the light phase in seconds)/(time outside)]	(Cummulative Spout zone duration during hours 8,9 and 10) > 0 and (Cummulative Spout zone duration during hours 11 and 12) > 0	Change in Spout zone during the last 5 hours of the light phase in anticipation of the upcoming dark phase
101	Spout zone change in response to dark	Anticipation / response to phase transition	None	fraction of time outside	[(Spout zone duration during hours 11 and 12 of the dark phase in seconds)/(time outside)]-[(Spout zone duration during hours 8, 9 and 10 of the dark phase in seconds)/(time outside)]	(Cummulative Spout zone duration during hours 8,9 and 10) > 0 and (Cummulative Spout zone duration during hours 11 and 12) > 0	Change in Spout zone during the last 5 hours of the dark phase in anticipation of the upcoming light phase
102	Mean activity duration - dark	Activity bout	Log10	s		Number of occurances during dark phase 3 > 10	Mean duration per activity bout during the dark phase
103	Activity number - dark	Activity bout	Log10	frequency		none	Cummulative number of activity bouts during the dark phase
104	OnShelter zone number - dark	Activity bout	Log10	frequency		none	Cummulative number of visits to OnShelter zone during the dark phase
105	Activity duration - dark	Activity bout	None	s		none	Cummulative duration of activity during the dark phase
106	Feeding zone duration - dark	Activity bout	Log10	s		none	Cummulative duration in feeding zone during the dark phase
107	OnShelter zone duration - dark	Activity bout	Log10	s		none	Cummulative duration in OnShelter zone during the dark phase
108	Spout zone duration - dark	Activity bout	Log10	s		none	Cummulative duration in Spout zone during the dark phase
109	Mean activity duration - light	Activity bout	Log10	s		Number of occurances during light phase 3 > 10	Mean duration per activity bout during the light phase
110	Activity number - light	Activity bout	Log10	frequency		none	Cummulative number of activity bouts during the light phase
111	OnShelter zone number - light	Activity bout	Log10	frequency		none	Cummulative number of visits to OnShelter zone during the light phase
112	Activity duration - light	Activity bout	Log10	s		none	Cummulative duration of activity during the light phase
113	Feeding zone duration - light	Activity bout	None	s		none	Cummulative duration in feeding zone during the light phase
114	OnShelter zone duration - light	Activity bout	Log10	cm		none	Cummulative duration in OnShelter zone during the light phase
115	Spout zone duration - light	Activity bout	Log10	s		none	Cummulative duration in Spout zone during the light phase
