# Supplementary material for: Sheltering Behavior and Locomotor Activity in 11 Genetically Diverse Common Inbred Mouse Strains Using Home-Cage Monitoring
Source: PLoS One. 2014 Sep 29;9(9):e108563. doi: 10.1371/journal.pone.0108563 (PMC4180925; doi:10.1371/journal.pone.0108563)
Supplement: Table S2 — Number of outside sleepers per strain. (PDF) [file pone.0108563.s002.pdf]

Strain	Total	Outside sleeper
129S1/SvImJ	61	2
A/J	49	6
BALB/cByJ	47	3
C3H/HeJ	29	-
C57BL/6J	112	5
CAST/EiJ	14	-
DBA/2J	40	1
FVB/NJ	49	12
NOD/LtJ	46	8
PWK/PhJ	15	-
WSB/EiJ	14	-
