# Supplementary material for: Sheltering Behavior and Locomotor Activity in 11 Genetically Diverse Common Inbred Mouse Strains Using Home-Cage Monitoring
Source: PLoS One. 2014 Sep 29;9(9):e108563. doi: 10.1371/journal.pone.0108563 (PMC4180925; doi:10.1371/journal.pone.0108563)
Supplement: Table S3 — Test statistics per measure. (PDF) [file pone.0108563.s003.pdf]

NR	Name	Segment	Units	Df between Groups	Df within Groups	F	ANOVA p-value	Bonferroni correction (P < 4.35E-4)	Genetic effect size	Lowest scoring strain	Highest scoring strain	Pairwise comparison with C57BL/6J (Sign. Tukey HSD) 129S1/SvImJ	A/J	BALB/cByJ	C3H/HeJ	CAST/EiJ	DBA/2J	FVB/NJ	NOD/LtJ	PWK/PhJ	WSB/EiJ
1	Long movement threshold	Move	cm	10	426	42.695	0.000	Sign.	0.35	C3H/HeJ	NOD/LtJ	0.000	0.000	0.027	0.000	0.277	0.000	0.000	0.005	0.000	0.924
2	Long movement max. velocity	Move	cm/s	10	426	47.182	0.000	Sign.	0.38	A/J	C57BL/6J	0.000	0.000	0.000	0.000	0.213	0.000	0.000	0.000	0.955	0.276
3	Long movement fraction of total movement	Move	fraction	10	426	26.113	0.000	Sign.	0.25	NOD/LtJ	A/J	0.000	0.000	0.325	0.005	0.001	0.000	0.002	0.000	0.000	0.024
4	Long movement distance - dark	Move	cm	10	426	97.379	0.000	Sign.	0.56	C3H/HeJ	BALB/c	0.000	1.000	0.000	0.000	0.534	0.000	0.000	1.000	1.000	0.962
5	Long movement distance - light	Move	cm	10	426	41.413	0.000	Sign.	0.35	DBA/2J	FVB/NJ	0.905	0.000	0.684	0.240	0.000	0.000	0.000	0.004	1.000	1.000
6	Long movement number - dark	Move	frequency	10	426	77.242	0.000	Sign.	0.50	C3H/HeJ	BALB/c	0.000	0.000	0.000	0.000	1.000	0.000	0.001	0.976	1.000	1.000
7	Long movement number - light	Move	frequency	10	426	33.033	0.000	Sign.	0.30	DBA/2J	FVB/NJ	1.000	0.000	0.195	1.000	0.000	0.000	0.000	0.279	0.991	1.000
8	Mean long movement distance - dark	Move	cm	10	426	45.788	0.000	Sign.	0.37	C3H/HeJ	NOD/LtJ	0.000	0.000	0.004	0.000	0.000	0.000	0.000	0.014	1.000	0.061
9	Mean long movement distance - light	Move	cm	10	424	47.319	0.000	Sign.	0.38	C3H/HeJ	NOD/LtJ	0.000	0.000	0.445	0.000	0.043	0.000	0.994	0.000	0.193	0.954
10	Mean short movement distance - dark	Move	cm	10	426	49.160	0.000	Sign.	0.39	C3H/HeJ	NOD/LtJ	0.000	0.000	0.996	0.000	0.944	0.000	0.001	0.000	0.000	1.000
11	Mean short movement distance - light	Move	cm	10	425	37.736	0.000	Sign.	0.33	DBA/2J	NOD/LtJ	0.000	0.000	1.000	0.000	1.000	0.000	0.684	0.000	0.000	1.000
12	Short movement distance - dark	Move	cm	10	426	125.699	0.000	Sign.	0.62	C3H/HeJ	NOD/LtJ	0.000	1.000	0.000	0.000	0.022	0.000	0.000	0.000	0.000	0.632
13	Short movement distance - light	Move	cm	10	426	38.833	0.000	Sign.	0.33	DBA/2J	FVB/NJ	0.000	0.000	0.004	0.002	0.039	0.000	0.000	0.000	0.648	0.808
14	Short movement number - dark	Move	frequency	10	426	121.568	0.000	Sign.	0.61	C3H/HeJ	BALB/c	0.000	0.020	0.000	0.000	0.011	0.000	0.007	0.000	0.035	0.209
15	Short movement number - light	Move	frequency	10	426	32.510	0.000	Sign.	0.29	DBA/2J	FVB/NJ	0.013	0.004	0.001	0.513	0.015	0.000	0.000	0.023	0.999	0.668
16	Long arrest threshold	Arrest	s	10	426	51.252	0.000	Sign.	0.40	BALB/c	PWK/PhJ	1.000	0.241	0.000	0.000	1.000	0.000	1.000	0.000	0.000	0.019
17	Long arrest duration - dark	Arrest	s	10	426	47.714	0.000	Sign.	0.38	FVB/NJ	PWK/PhJ	0.000	0.970	0.151	0.000	0.909	0.000	0.000	0.000	0.000	0.993
18	Long arrest duration - light	Arrest	s	10	426	24.964	0.000	Sign.	0.24	A/J	CAST/EiJ	0.447	0.000	0.805	0.000	0.000	0.000	0.084	0.977	1.000	0.883
19	Long arrest number - dark	Arrest	frequency	10	426	118.651	0.000	Sign.	0.61	C3H/HeJ	BALB/c	0.000	0.000	0.000	0.000	1.000	0.001	0.015	0.000	1.000	1.000
20	Long arrest number - light	Arrest	frequency	10	426	44.209	0.000	Sign.	0.36	DBA/2J	FVB/NJ	0.000	0.000	0.000	0.065	0.000	0.000	0.000	0.009	0.769	0.988
21	Mean long arrest duration - dark	Arrest	s	10	426	158.628	0.000	Sign.	0.67	BALB/c	C3H/HeJ	0.000	0.000	0.000	0.000	0.918	0.000	0.000	0.000	0.000	0.991
22	Mean long arrest duration - light	Arrest	s	10	414	60.900	0.000	Sign.	0.45	BALB/c	C3H/HeJ	0.199	0.000	0.000	0.000	1.000	0.000	0.000	0.000	0.997	0.672
23	Mean short arrest duration - dark	Arrest	s	10	426	34.891	0.000	Sign.	0.31	BALB/c	PWK/PhJ	0.980	0.746	0.000	0.000	0.875	0.000	0.141	0.018	0.000	0.251
24	Mean short arrest duration - light	Arrest	s	10	425	27.075	0.000	Sign.	0.26	BALB/c	PWK/PhJ	0.014	0.991	0.000	0.001	0.929	0.000	0.833	0.000	0.000	0.958
25	Short arrest duration - dark	Arrest	s	10	426	55.777	0.000	Sign.	0.42	C3H/HeJ	BALB/c	0.000	0.001	0.000	0.000	0.999	0.940	0.675	0.000	0.262	0.262
26	Short arrest duration - light	Arrest	s	10	426	28.827	0.000	Sign.	0.27	DBA/2J	FVB/NJ	0.199	0.003	0.611	1.000	0.000	0.000	0.000	0.925	0.888	0.960
27	Short arrest number - dark	Arrest	frequency	10	426	118.492	0.000	Sign.	0.61	C3H/HeJ	BALB/c	0.000	0.000	0.000	0.000	0.619	0.000	0.000	0.000	0.723	0.977
28	Short arrest number - light	Arrest	frequency	10	426	31.220	0.000	Sign.	0.28	DBA/2J	FVB/NJ	0.882	0.010	0.009	0.988	0.000	0.000	0.000	0.080	1.000	0.998
29	Long shelter visit threshold	Sheltering	s	10	426	16.196	0.000	Sign.	0.17	129S1/Sv	PWK/PhJ	0.000	0.000	0.001	0.910	0.358	0.000	0.452	0.045	1.000	0.029
30	Short shelter visit threshold	Sheltering	s	10	426	31.351	0.000	Sign.	0.28	BALB/c	C3H/HeJ	0.067	0.000	0.000	0.000	0.942	0.103	0.896	0.889	0.947	0.997
31	Long shelter visit duration - dark	Sheltering	s	10	426	33.973	0.000	Sign.	0.30	PWK/PhJ	FVB/NJ	0.000	0.901	1.000	0.000	0.403	0.998	0.000	1.000	0.000	0.965
32	Long shelter visit duration - light	Sheltering	s	10	426	20.568	0.000	Sign.	0.20	CAST/EiJ	DBA/2J	0.663	0.002	1.000	0.985	0.000	0.000	0.000	0.936	1.000	0.931
33	Long shelter visit fraction of total visits	Sheltering	fraction	10	426	106.969	0.000	Sign.	0.58	BALB/c	C3H/HeJ	0.000	0.001	0.000	0.000	0.999	0.000	0.999	0.999	1.000	1.000
34	Long shelter visit number - dark	Sheltering	frequency	10	426	14.657	0.000	Sign.	0.15	PWK/PhJ	129S1/Sv	0.000	0.464	0.110	0.997	1.000	0.004	0.633	0.973	0.000	0.514
35	Long shelter visit number - light	Sheltering	frequency	10	426	18.94392	0.000	Sign.	0.19	DBA/2J	NOD/LtJ	0.000	0.000	0.000	0.000	1.000	0.000	0.630	0.347	0.801	0.995
36	Mean long shelter visit duration	Sheltering	s	10	426	15.129	0.000	Sign.	0.16	NOD/LtJ	BALB/c	0.000	0.950	0.000	0.142	1.000	1.000	0.411	0.000	0.140	0.079
37	Mean short shelter visit duration - dark	Sheltering	s	10	409	33.003	0.000	Sign.	0.30	BALB/c	C3H/HeJ	0.530	0.000	0.000	0.000	0.851	0.053	1.000	0.265	1.000	1.000
38	Mean short shelter visit duration - light	Sheltering	s	10	313	17.104	0.000	Sign.	0.23	BALB/c	C3H/HeJ	0.000	0.000	0.000	0.632	0.808	0.981	1.000	0.006	0.884	0.997
39	Short shelter visit duration - dark	Sheltering	s	10	426	8.521	0.000	Sign.	0.09	C3H/HeJ	WSB/EiJ	0.000	0.578	0.278	0.000	0.820	0.000	0.121	0.079	0.035	0.997
40	Short shelter visit duration - light	Sheltering	s	10	426	10.249	0.000	Sign.	0.11	C3H/HeJ	FVB/NJ	0.000	0.633	0.402	0.000	1.000	0.000	0.403	0.994	0.699	0.668
41	Short shelter visit number - dark	Sheltering	frequency	10	426	64.209	0.000	Sign.	0.45	C3H/HeJ	BALB/c	0.000	0.714	0.000	0.000	0.006	0.000	0.006	0.997	0.011	1.000
42	Short shelter visit number - light	Sheltering	frequency	10	426	27.357	0.000	Sign.	0.26	C3H/HeJ	NOD/LtJ	0.034	0.597	0.100	0.000	1.000	0.000	0.080	0.010	0.104	0.934
		**Temporal aspect**																			
43	Activity duration - habituation ratio dark	Habituation	ratio	10	426	6.555	0.000	Sign.	0.07	A/J	C57BL/6J	0.034	0.000	0.003	0.005	0.547	0.003	0.000	0.017	0.162	0.989
44	Feeding zone duration - habituation ratio dark	Habituation	ratio	10	426	3.455	0.000	Sign.	0.03	C57BL/6J	PWK/PhJ	1.000	0.966	0.144	0.964	0.962	0.678	0.997	0.973	0.001	0.012
45	Long shelter visit duration - habituation ratio dark	Habituation	ratio	10	393	8.905	0.000	Sign.	0.10	WSB/EiJ	A/J	0.062	0.000	0.000	0.115	0.172	0.966	0.000	0.666	1.000	1.000
46	OnShelter zone duration - habituation ratio dark	Habituation	ratio	10	360	22.237	0.000	Sign.	0.25	C3H/HeJ	C57BL/6J	0.000	0.000	0.068	0.000	0.013	0.003	0.000	0.000	0.000	0.000
47	Mean short movement distance - habituation ratio dark	Habituation	ratio	10	426	4.189	0.000	Sign.	0.04	A/J	C57BL/6J	0.989	0.000	0.084	0.041	1.000	0.001	0.038	0.064	0.965	0.887
48	Mean activity duration - habituation ratio dark	Habituation	ratio	10	426	4.134	0.000	Sign.	0.04	A/J	129S1/Sv	0.222	0.005	0.641	1.000	0.991	0.986	1.000	0.744	1.000	1.000
49	Activity number - habituation ratio dark	Habituation	ratio	10	426	7.598	0.000	Sign.	0.08	129S1/Sv	C57BL/6J	0.000	0.001	0.094	0.000	0.912	0.002	0.000	0.371	0.052	0.748
50	Mean short arrest duration - habituation ratio dark	Habituation	ratio	10	426	27.701	0.000	Sign.	0.26	C3H/HeJ	A/J	0.000	0.000	0.000	0.000	1.000	0.001	0.000	0.004	0.986	0.938
51	Long arrest duration - habituation ratio dark	Habituation	ratio	10	426	8.471	0.000	Sign.	0.09	FVB/NJ	PWK/PhJ	1.000	0.608	1.000	1.000	0.999	1.000	0.000	0.874	0.383	0.745
52	Mean long arrest duration - habituation ratio dark	Habituation	ratio	10	426	14.371	0.000	Sign.	0.15	FVB/NJ	PWK/PhJ	0.005	0.457	0.375	0.496	1.000	0.996	0.000	0.433	0.000	0.089
53	Long arrest number - habituation ratio dark	Habituation	ratio	10	426	4.463	0.000	Sign.	0.04	129S1/Sv	BALB/c	0.005	1.000	0.465	0.431	0.997	1.000	0.354	1.000	0.592	0.999
54	Mean short shelter visit duration - habituation ratio dark	Habituation	ratio	10	400	3.448	0.000	Sign.	0.03	PWK/PhJ	NOD/LtJ	0.935	0.997	1.000	0.785	1.000	1.000	0.970	0.036	0.415	0.580
55	Mean long movement distance - habituation ratio dark	Habituation	ratio	10	426	10.621	0.000	Sign.	0.11	DBA/2J	129S1/Sv	0.000	0.986	0.983	0.033	1.000	0.003	0.004	0.756	0.214	0.717
56	Spout zone duration - habituation ratio dark	Habituation	ratio	10	426	4.880	0.000	Sign.	0.05	A/J	C57BL/6J	0.002	0.000	0.039	0.999	0.890	0.014	0.002	0.002	0.087	0.146
57	Activity duration - habituation ratio light	Habituation	ratio	10	426	6.189	0.000	Sign.	0.06	BALB/c	WSB/EiJ	0.521	0.997	0.492	0.432	1.000	0.008	1.000	1.000	0.606	0.000
58	Feeding zone duration - habituation ratio light	Habituation	ratio	10	420	6.079	0.000	Sign.	0.06	NOD/LtJ	WSB/EiJ	0.957	1.000	1.000	0.985	1.000	0.692	1.000	1.000	0.999	0.000
59	Long shelter visit duration - habituation ratio light	Habituation	ratio	10	423	4.748	0.000	Sign.	0.05	WSB/EiJ	FVB/NJ	0.811	1.000	0.980	0.693	1.000	0.824	0.003	1.000	0.810	0.272
60	OnShelter zone duration - habituation ratio light	Habituation	ratio	10	279	4.894	0.000	Sign.	0.07	BALB/c	WSB/EiJ	0.353	1.000	0.000	0.948	0.999	1.000	0.933	0.997	0.999	0.041
61	Mean short movement distance - habituation ratio light	Habituation	ratio	10	425	2.648	0.004	Non-Sign.	0.02	BALB/c	C3H/HeJ	1.000	0.629	0.055	0.998	1.000	1.000	1.000	0.142	1.000	0.985
62	Mean activity duration - habituation ratio light	Habituation	ratio	10	406	3.467	0.000	Sign.	0.03	BALB/c	DBA/2J	0.451	1.000	0.879	0.925	1.000	0.003	1.000	1.000	0.952	0.998
63	Activity number - habituation ratio light	Habituation	ratio	10	414	5.636	0.000	Sign.	0.06	BALB/c	WSB/EiJ	0.999	0.998	0.856	0.379	0.962	0.951	1.000	0.921	0.804	0.000
64	Mean short arrest duration - habituation ratio light	Habituation	ratio	10	425	9.831	0.000	Sign.	0.10	129S1/Sv	BALB/c	0.551	0.000	0.000	1.000	0.978	0.253	0.002	0.079	1.000	1.000
65	Long arrest duration - habituation ratio light	Habituation	ratio	10	422	5.023	0.000	Sign.	0.05	FVB/NJ	WSB/EiJ	0.682	0.830	1.000	0.995	1.000	0.984	0.397	0.999	0.888	0.000
66	Mean long arrest duration - habituation ratio light	Habituation	ratio	10	406	3.708	0.000	Sign.	0.04	FVB/NJ	WSB/EiJ	0.985	0.879	0.664	0.971	0.994	1.000	0.002	0.045	1.000	1.000
67	Long arrest number - habituation ratio light	Habituation	ratio	10	411	3.588	0.000	Sign.	0.03	FVB/NJ	WSB/EiJ	0.999	0.619	0.888	0.406	0.960	0.894	1.000	0.881	0.424	0.000
68	Mean short shelter visit duration - habituation ratio light	Habituation	ratio	9	269	1.016	0.428	Non-Sign.	0.00	WSB/EiJ	CAST/EiJ	NA	NA	NA	NA	NA	NA	NA	NA	NA	NA
69	Mean long movement distance - habituation ratio light	Habituation	ratio	10	424	5.410	0.000	Sign.	0.05	DBA/2J	129S1/Sv	0.001	1.000	0.998	0.770	1.000	0.050	1.000	0.992	0.793	1.000
70	Spout zone duration - habituation ratio light	Habituation	ratio	10	364	3.479	0.000	Sign.	0.04	BALB/c	WSB/EiJ	0.844	1.000	0.059	0.642	1.000	1.000	0.888	1.000	0.996	0.265
71	Activity duration - darklight index	DarkLightIndex	index	10	426	29.209	0.000	Sign.	0.27	FVB/NJ	A/J	0.000	0.220	0.986	0.000	0.000	1.000	0.000	0.910	1.000	1.000
72	Long shelter visit duration - darklight index	DarkLightIndex	index	10	426	31.903	0.000	Sign.	0.29	PWK/PhJ	FVB/NJ	0.000	1.000	1.000	0.000	0.001	1.000	0.000	1.000	0.000	0.801
73	Mean short movement distance - darklight index	DarkLightIndex	index	10	425	12.030	0.000	Sign.	0.13	129S1/Sv	NOD/LtJ	0.000	0.975	1.000	0.000	0.039	0.001	0.000	1.000	0.002	1.000
74	Feeding zone duration - darklight index	DarkLightIndex	index	10	426	51.190	0.000	Sign.	0.40	FVB/NJ	A/J	1.000	0.000	0.992	0.444	0.000	0.000	0.000	0.994	0.001	0.737
75	Mean activity duration - darklight index	DarkLightIndex	index	10	412	9.775	0.000	Sign.	0.11	DBA/2J	BALB/c	0.000	0.838	0.990	0.001	0.999	0.000	0.000	0.994	0.010	1.000
76	Activity number - darklight index	DarkLightIndex	index	10	426	54.738	0.000	Sign.	0.41	FVB/NJ	DBA/2J	0.727	0.000	0.943	0.000	0.000	0.000	0.000	0.494	0.856	1.000
77	Mean short arrest duration - darklight index	DarkLightIndex	index	10	425	10.412	0.000	Sign.	0.11	WSB/EiJ	129S1/Sv	0.000	0.997	0.574	0.748	1.000	0.101	0.906	0.011	1.000	0.563
78	Long arrest duration - darklight index	DarkLightIndex	index	10	426	41.896	0.000	Sign.	0.35	FVB/NJ	DBA/2J	1.000	0.000	1.000	0.522	0.000	0.000	0.000	0.997	0.001	0.635
79	Mean long arrest duration - darklight index	DarkLightIndex	index	10	414	5.834	0.000	Sign.	0.06	BALB/c	PWK/PhJ	0.967	0.690	1.000	0.008	1.000	0.997	0.341	1.000	0.000	0.144
80	Long arrest number - darklight index	DarkLightIndex	index	10	426	41.649	0.000	Sign.	0.35	FVB/NJ	DBA/2J	1.000	0.000	1.000	0.000	0.000	0.000	0.000	0.988	0.694	1.000
81	Mean short shelter visit duration - darklight index	DarkLightIndex	index	10	312	6.144	0.000	Sign.	0.09	PWK/PhJ	C3H/HeJ	0.061	0.003	0.981	0.034	1.000	0.320	0.995	0.193	0.379	0.221
82	Long shelter visit number - darklight index	DarkLightIndex	index	10	426	23.383	0.000	Sign.	0.23	PWK/PhJ	129S1/Sv	0.000	0.000	1.000	0.015	1.000	0.000	0.998	1.000	0.000	0.931
83	Mean long movement distance - darklight index	DarkLightIndex	index	10	424	12.668	0.000	Sign.	0.13	FVB/NJ	PWK/PhJ	0.990	0.000	0.726	0.599	0.941	0.246	0.000	0.001	0.002	0.563
84	OnShelter zone duration - darklight index	DarkLightIndex	index	10	379	48.374	0.000	Sign.	0.41	FVB/NJ	A/J	0.960	0.783	1.000	0.000	0.000	0.991	0.000	0.000	0.786	0.014
85	Spout zone duration - darklight index	DarkLightIndex	index	10	426	44.730	0.000	Sign.	0.36	FVB/NJ	A/J	1.000	0.000	0.690	0.000	0.000	0.000	0.000	0.136	1.000	1.000
86	Activity change in anticipation of light	Pattern	fraction of total time	10	426	14.219	0.000	Sign.	0.15	BALB/c	C57BL/6J	0.000	0.063	0.000	0.002	0.972	0.000	0.000	0.002	0.000	0.003
87	Activity change in response to to light	Pattern	fraction of total time	10	426	10.916	0.000	Sign.	0.12	BALB/c	FVB/NJ	1.000	0.405	0.000	0.159	0.995	0.978	0.000	1.000	0.975	0.977
88	Activity change in anticipation of dark	Pattern	fraction of total time	10	426	8.314	0.000	Sign.	0.09	DBA/2J	CAST/EiJ	1.000	0.516	1.000	0.951	0.000	0.590	0.000	0.994	0.998	0.886
89	Activity change in response to to dark	Pattern	fraction of total time	10	426	28.321	0.000	Sign.	0.26	C3H/HeJ	BALB/c	0.000	0.999	0.002	0.000	1.000	0.000	0.000	0.315	1.000	1.000
90	Feeding zone change in anticipation light	Pattern	fraction of time outside	10	408	0.827	0.603	Non-Sign.	0.00	WSB/EiJ	BALB/c	1.000	1.000	0.817	1.000	1.000	0.995	1.000	0.999	1.000	0.999
91	Feeding zone change in response to light	Pattern	fraction of time outside	10	309	3.859	0.000	Sign.	0.05	A/J	CAST/EiJ	0.865	0.153	0.485	0.990	0.442	0.928	0.113	1.000	1.000	1.000
92	Feeding zone change in anticipation dark	Pattern	fraction of time outside	10	377	1.290	0.234	Non-Sign.	0.00	CAST/EiJ	A/J	0.999	0.965	1.000	1.000	0.897	1.000	0.588	1.000	0.996	1.000
93	Feeding zone change in response to dark	Pattern	fraction of time outside	10	388	5.428	0.000	Sign.	0.06	C57BL/6J	C3H/HeJ	0.909	0.005	0.999	0.000	0.399	0.058	0.444	0.002	0.363	0.344
94	OnShelter zone change in anticipation light	Pattern	fraction of time outside	10	408	2.650	0.004	Non-Sign.	0.02	BALB/c	FVB/NJ	0.985	0.956	0.055	1.000	0.818	1.000	0.284	1.000	1.000	1.000
95	OnShelter zone change in response to light	Pattern	fraction of time outside	10	309	9.126	0.000	Sign.	0.13	BALB/c	NOD/LtJ	0.003	0.081	1.000	0.000	0.777	0.187	0.000	0.000	0.003	0.069
96	OnShelter zone change in anticipation dark	Pattern	fraction of time outside	10	377	1.630	0.096	Non-Sign.	0.01	PWK/PhJ	FVB/NJ	0.997	1.000	1.000	1.000	0.999	0.915	0.953	1.000	0.282	1.000
97	OnShelter zone change in response to dark	Pattern	fraction of time outside	10	388	14.778	0.000	Sign.	0.17	PWK/PhJ	C57BL/6J	0.000	0.032	1.000	0.000	0.000	0.397	0.000	0.000	0.000	0.000
98	Spout zone change in anticipation light	Pattern	fraction of time outside	10	408	0.825	0.605	Non-Sign.	0.00	PWK/PhJ	NOD/LtJ	1.000	1.000	1.000	1.000	1.000	1.000	0.997	0.857	0.997	0.998
99	Spout zone change in response to light	Pattern	fraction of time outside	10	309	1.684	0.084	Non-Sign.	0.01	PWK/PhJ	NOD/LtJ	1.000	1.000	1.000	1.000	1.000	1.000	0.929	0.167	0.952	0.997
100	Spout zone change in anticipation dark	Pattern	fraction of time outside	10	377	1.308	0.224	Non-Sign.	0.00	A/J	PWK/PhJ	1.000	0.945	1.000	1.000	1.000	0.955	0.950	1.000	0.248	1.000
101	Spout zone change in response to dark	Pattern	fraction of time outside	10	388	2.474	0.007	Non-Sign.	0.02	WSB/EiJ	PWK/PhJ	0.999	1.000	1.000	0.931	0.717	0.999	0.961	0.582	0.934	0.332
102	Mean activity duration - dark	Activity bout	s	10	426	14.749	0.000	Sign.	0.15	129S1/Sv	FVB/NJ	0.000	0.030	0.014	1.000	1.000	0.868	0.000	0.893	0.969	0.656
103	Activity number - dark	Activity bout	frequency	10	426	85.483	0.000	Sign.	0.53	C3H/HeJ	BALB/c	0.000	0.000	0.000	0.000	1.000	0.000	0.000	1.000	0.730	0.371
104	OnShelter zone number - dark	Activity bout	frequency	10	426	93.940	0.000	Sign.	0.55	129S1/Sv	BALB/c	0.000	0.000	1.000	0.000	0.575	0.000	0.000	0.000	0.090	0.985
105	Activity duration - dark	Activity bout	s	10	426	54.972	0.000	Sign.	0.41	C3H/HeJ	BALB/c	0.000	0.554	0.000	0.000	1.000	0.000	0.000	0.996	0.018	0.993
106	Feeding zone duration - dark	Activity bout	s	10	426	35.045	0.000	Sign.	0.31	129S1/Sv	DBA/2J	0.000	0.000	0.000	0.000	0.259	0.000	0.000	0.000	0.010	0.336
107	OnShelter zone duration - dark	Activity bout	s	10	426	45.243	0.000	Sign.	0.37	129S1/Sv	BALB/c	0.000	0.000	1.000	0.000	0.872	0.983	0.399	0.000	0.908	0.496
108	Spout zone duration - dark	Activity bout	s	10	426	29.792	0.000	Sign.	0.27	FVB/NJ	PWK/PhJ	0.000	1.000	0.175	0.000	0.981	0.000	0.000	0.000	0.000	0.999
109	Mean activity duration - light	Activity bout	s	10	412	13.033	0.000	Sign.	0.14	BALB/c	FVB/NJ	0.463	1.000	0.118	0.002	1.000	0.000	0.000	0.828	0.002	1.000
110	Activity number - light	Activity bout	frequency	10	426	40.041	0.000	Sign.	0.34	DBA/2J	CAST/EiJ	0.036	0.000	0.107	0.134	0.000	0.000	0.000	0.846	1.000	1.000
111	OnShelter zone number - light	Activity bout	frequency	10	426	70.747	0.000	Sign.	0.48	129S1/Sv	FVB/NJ	0.000	0.000	0.007	0.000	0.000	0.000	0.000	0.949	0.972	1.000
112	Activity duration - light	Activity bout	s	10	426	21.645	0.000	Sign.	0.21	DBA/2J	FVB/NJ	0.970	0.000	1.000	1.000	0.000	0.000	0.000	0.543	0.785	1.000
113	Feeding zone duration - light	Activity bout	s	10	426	41.710	0.000	Sign.	0.35	A/J	CAST/EiJ	0.193	0.000	0.487	0.000	0.000	0.143	0.000	0.223	0.349	0.999
114	OnShelter zone duration - light	Activity bout	cm	10	426	55.923	0.000	Sign.	0.42	129S1/Sv	FVB/NJ	0.000	0.000	0.144	0.065	0.029	0.000	0.000	0.560	1.000	1.000
115	Spout zone duration - light	Activity bout	s	10	426	34.809	0.000	Sign.	0.31	A/J	CAST/EiJ	0.000	0.000	0.094	1.000	0.011	0.000	0.018	1.000	0.957	1.000

NR	Strain average 129S1/SvI	A/J	BALB/cByJ	C3H/HeJ	C57BL/6J	CAST/EiJ	DBA/2J	FVB/NJ	NOD/LtJ	PWK/PhJ	WSB/EiJ
1	1.267892	1.387717	1.652838	0.991547	1.878111	1.614233	1.166435	1.439779	2.177708	1.319855	1.721603
2	17.93782	13.92651	18.10615	14.61253	21.05788	19.159	16.52427	17.45317	18.60651	20.09418	19.24213
3	0.459839	0.474067	0.42865	0.448233	0.405638	0.471381	0.453579	0.447432	0.325387	0.472582	0.456923
4	12769.68	28248.29	46566.82	6030.434	28490.58	23138.57	15215.03	18983.94	29883.38	29432.56	25047.8
5	2612.996	1106.348	4110.971	2083.289	3156.879	8752.36	721.9988	12673.56	5604.669	3383.263	3363.93
6	1110.069	2546.826	3419.739	691.2448	1847.476	1899.274	1327.897	1439.459	1722.001	1899.718	1899.696
7	232.8619	117.5158	302.7213	236.3332	214.6689	713.4954	70.80362	896.5621	302.5565	266.0615	247.2908
8	11.52258	11.10224	13.62912	8.737224	15.42992	12.19215	11.46467	13.21925	17.37496	15.50016	13.20138
9	11.27485	9.556553	13.60133	8.826762	14.72833	12.27778	10.24471	14.16757	18.54993	12.72377	13.65094
10	1.016801	1.162625	1.310557	0.917289	1.347164	1.264493	0.927123	1.179344	1.674223	0.98663	1.371709
11	1.028041	1.10365	1.231383	0.915299	1.256484	1.254532	0.91504	1.185377	1.559731	0.990052	1.28669
12	1456.939	3920.735	7366.924	912.5694	3790.487	2642.341	1824.762	2695.796	8193.259	2141.202	3076.258
13	323.1872	315.3066	963.4294	314.3095	582.5233	1138.868	138.8825	1637.645	1113.478	393.282	408.7467
14	1439.687	3387.574	5643.324	998.905	2829.597	2100.698	1971.667	2298.569	4934.996	2177.742	2279.517
15	315.781	290.1367	785.9602	344.5919	465.6256	912.8318	152.2945	1390.221	720.5102	398.2701	322.1956
16	5.027887	4.589597	3.273761	6.544206	5.079374	5.140757	7.3209	5.049824	3.589517	7.729463	4.084811
17	8795.786	11722.16	9554.273	14903.77	10991.57	12396.18	15343.6	5679.788	8374.65	22095.26	11954.08
18	2195.921	1224.914	2284.433	4769.29	2784.391	7171.029	1425.272	3719.533	2407.401	2671.289	2063.521
19	290.8578	722.6046	979.0071	176.107	461.8333	460.6528	373.8795	385.1742	669.7509	457.3117	459.8356
20	50.5511	46.07299	172.6958	57.99419	89.24638	223.2373	22.90722	243.9676	142.3938	64.32789	72.15801
21	30.30279	16.24154	9.775541	84.69614	23.82129	26.96093	41.07947	14.79411	12.57172	48.38119	26.06684
22	34.20001	17.02778	12.14564	74.99792	28.5089	30.92807	43.99081	14.71751	14.70663	31.80912	22.46282
23	1.014108	0.92598	0.765334	1.197022	0.980983	1.058902	1.309114	1.074756	0.870971	1.32998	0.861658
24	0.91983	0.987039	0.833256	1.190853	1.022612	1.098958	1.282194	1.082318	0.833518	1.359736	0.95475
25	2522.262	5210.491	6549.421	1894.709	4330.422	4072.749	4029.223	3905.393	5617.049	5130.209	3505.593
26	478.9904	394.9211	805.8191	633.8635	635.7747	1604.999	266.4115	2262.026	762.9314	842.4207	497.038
27	2360.838	5431.937	8415.93	1543.412	4356.333	3735.841	2997.172	3464.351	6209.979	3794.78	3966.099
28	527.164	401.3426	973.6191	534.837	625.1368	1467.828	210.0287	2103.591	918.5993	622.9725	525.2704
29	9.094496	9.163356	9.809482	10.18409	10.44466	9.884617	9.567924	10.08706	9.936042	10.51718	9.653116
30	4.025451	3.478327	2.86582	6.428652	4.526745	4.92581	5.075893	4.824105	4.228948	4.908781	4.795587
31	24962.21	16665.92	15242.64	21790.31	15190.94	18706.38	16103.47	25906.13	14971.8	6897.863	13217.17
32	38071.05	39558.61	37397.45	36152.82	36984.74	30294.25	40023.38	31912.41	36052.8	37170.67	38393.03
33	0.11916	0.026718	0.016115	0.286271	0.055496	0.064515	0.112274	0.061182	0.049834	0.058328	0.062725
34	9.648694	6.20458	3.575345	5.513391	4.911903	4.33781	7.4931	6.141365	5.65136	1.968918	6.992628
35	4.315216	3.624292	4.245076	4.078722	5.615419	5.243976	3.220958	6.376138	6.52905	4.777495	6.173608
36	4243.156	5520.809	6627.726	6049.245	5166.277	4888.996	5232.471	4587.185	3921.221	6361.98	4101.102
37	5.759383	3.983343	2.357801	23.70521	6.96551	8.891632	9.54349	7.358836	5.364747	7.367618	7.276016
38	4.347127	3.628892	2.828023	14.25271	7.162969	9.209822	8.530537	7.385134	4.873929	9.263702	6.067314
39	441.7941	772.8713	739.7955	430.2354	934.0425	725.1914	532.1357	695.1222	684.458	540.6338	1050.101
40	60.10283	104.525	97.57814	40.87912	163.7741	185.2951	42.94259	285.2454	209.1639	85.1353	82.10537
41	46.43579	154.3932	263.5624	13.10216	125.8086	65.48069	44.32108	80.80833	112.681	68.42054	120.1721
42	13.53604	32.388	37.62104	2.52236	22.79539	20.41434	5.552989	39.65128	44.32443	10.33129	15.38518
43	0.838198	0.668506	0.791134	0.765798	0.989585	0.821339	0.781705	0.719956	0.802379	0.778829	0.89907
44	1.085563	1.131273	1.227123	1.145157	1.051231	1.181064	1.176121	1.113399	1.133025	1.494147	1.428517
45	1.034244	1.338957	1.251191	1.072497	0.859205	1.132809	0.944859	1.268869	0.991025	0.834269	0.828372
46	0.504994	0.865627	1.23644	0.408907	1.641327	0.945658	1.117974	0.595456	0.540662	0.733753	0.621869
47	1.009781	0.981602	0.993355	0.986896	1.018093	1.015518	0.98227	0.989185	0.990939	1.001642	0.997685
48	1.032698	0.780183	0.853521	0.902973	0.933021	0.864199	0.884524	0.90782	0.854771	0.906853	0.983408
49	0.800368	0.881478	0.933538	0.841238	1.057543	0.957608	0.882607	0.806131	0.951983	0.857402	0.936469
50	0.952037	1.150675	1.107276	0.915311	1.032008	1.041626	1.110177	1.146556	1.102621	1.002648	0.994769
51	1.073702	0.959457	1.060244	1.046856	1.083799	1.168372	1.126365	0.65053	0.979995	1.322916	1.274123
52	1.28624	0.950399	0.944946	1.228336	1.076211	1.087506	1.135109	0.723281	0.941566	1.539922	1.36814
53	0.851758	1.016889	1.125274	0.889404	1.014333	1.088961	0.995091	0.893401	1.027365	0.866809	0.951374
54	0.940699	0.95858	0.981488	0.883926	1.000636	1.030623	1.003351	0.94101	1.161992	0.850193	0.860706
55	1.090921	0.988462	1.029697	1.078717	1.008792	1.009191	0.936483	1.084509	0.974895	1.082058	1.062166
56	0.762982	0.703755	0.791253	0.939913	0.999668	0.84009	0.763738	0.722156	0.727366	0.711876	0.721606
57	0.972494	0.868672	0.579687	1.049526	0.776778	0.814892	1.191488	0.754341	0.84103	1.101918	1.763885
58	1.08317	0.91832	0.997163	1.097311	0.959421	1.010834	1.171178	0.89756	0.891987	1.08376	2.489477
59	1.001012	1.025156	1.064243	0.985877	1.036412	1.028971	0.99581	1.139872	1.044477	0.976522	0.946528
60	0.378185	0.862919	0.367123	0.631667	0.807257	0.935569	0.862833	0.658147	0.701936	0.92939	1.487576
61	0.996812	0.97618	0.961566	1.01715	1.002876	1.001055	1.011917	0.999162	0.963674	1.008213	0.978836
62	1.092573	0.970856	0.808936	1.063591	0.927228	0.841926	1.35387	0.906855	0.884458	1.09634	1.047175
63	0.882194	0.8896	0.720172	1.024097	0.827771	0.974926	0.936444	0.846643	0.937371	1.018034	1.708802
64	0.922251	1.107507	1.116918	0.95555	0.96911	1.017851	1.036745	1.081991	1.051018	0.958526	0.988017
65	1.158674	1.155037	0.970778	1.089617	0.95012	1.062529	1.09702	0.685577	0.856187	1.243675	2.237468
66	1.254062	1.015024	0.990602	1.02961	1.157387	1.016326	1.145356	0.800666	0.872504	1.059868	1.270017
67	0.920064	1.057039	1.004862	1.124935	0.851282	1.061693	1.02784	0.838844	1.017226	1.215068	1.867136
68	0.953312	1.005583	1.023773	NA	0.941924	1.102207	0.957996	1.000091	1.072367	0.948408	0.843645
69	1.128173	1.02515	0.998158	0.967399	1.022205	1.023807	0.934848	1.02649	0.992387	0.951864	1.03371
70	0.694011	0.787439	0.548655	1.130519	0.85662	0.853925	0.823003	0.684706	0.930913	1.026295	1.382224
71	0.798781	0.920244	0.893062	0.72719	0.872227	0.731098	0.881063	0.622363	0.843124	0.861182	0.86904
72	0.392662	0.291494	0.285963	0.373431	0.284868	0.380979	0.279438	0.445527	0.278558	0.155677	0.246889
73	0.497194	0.513411	0.515708	0.500412	0.517061	0.502037	0.503725	0.498904	0.517881	0.49893	0.515426
74	0.800378	0.920651	0.811792	0.757164	0.796013	0.645214	0.906327	0.579409	0.8122	0.892116	0.839112
75	0.492771	0.558766	0.606867	0.502846	0.588155	0.56598	0.473592	0.506217	0.569405	0.491015	0.579907
76	0.815224	0.912942	0.855737	0.73342	0.837345	0.675816	0.919258	0.618309	0.806738	0.870439	0.840568
77	0.524058	0.484906	0.478927	0.501307	0.489959	0.490243	0.506312	0.498631	0.510597	0.493577	0.472322
78	0.810829	0.906809	0.809987	0.76425	0.802262	0.640058	0.914244	0.608445	0.787246	0.900289	0.850115
79	0.477477	0.490189	0.447332	0.529194	0.458331	0.466099	0.47547	0.500407	0.461252	0.60271	0.533035
80	0.826858	0.919157	0.83425	0.743058	0.825906	0.672613	0.922001	0.608434	0.807579	0.871	0.836578
81	0.528793	0.540918	0.469157	0.653307	0.485412	0.492214	0.538467	0.499482	0.522659	0.430057	0.550269
82	0.682184	0.615868	0.455313	0.568489	0.465963	0.453811	0.672574	0.489796	0.46763	0.294248	0.522538
83	0.505681	0.53869	0.500511	0.497362	0.511638	0.498276	0.527873	0.482673	0.483583	0.548974	0.491796
84	0.962877	0.972002	0.946078	0.753032	0.933728	0.638227	0.954321	0.635211	0.790941	0.88417	0.829775
85	0.859921	0.953653	0.895483	0.781531	0.865166	0.688614	0.943213	0.61838	0.817419	0.879944	0.850489
86	0.006955	0.044557	-0.08994	0.011452	0.107382	0.067403	0.011718	-0.05935	0.02078	-0.04394	-0.01849
87	-0.07569	-0.11637	-0.18773	0.005264	-0.06541	-0.03053	-0.09314	0.061899	-0.06116	-0.10713	-0.10806
88	0.005071	0.037612	0.004687	-0.01223	0.008907	0.127325	-0.01942	0.080068	0.023384	0.026726	0.041771
89	0.114904	0.289362	0.35516	0.045042	0.27309	0.288083	0.137955	0.162922	0.221077	0.272139	0.290222
90	-0.03348	-0.0347	0.031363	-0.05306	-0.03721	-0.04502	-0.07751	-0.01899	-0.07319	-0.07896	-0.08876
91	-0.07217	-0.32333	-0.02357	-0.09259	-0.15825	0.011851	-0.25422	-0.00801	-0.16458	-0.11257	-0.08781
92	0.027694	0.146028	0.078646	0.0952	0.06464	-0.05025	0.082565	-0.03955	0.059068	-0.00656	0.092794
93	-0.15572	0.009364	-0.18324	0.070503	-0.21927	-0.05304	-0.05759	-0.1097	-0.02415	-0.05425	-0.03483
94	-0.00611	-0.00937	-0.02892	0.002228	0.004665	-0.02315	0.000784	0.034727	0.002986	0.005977	-0.00173
95	-0.01865	-0.02726	-0.08121	0.004917	-0.07296	-0.03886	-0.02742	-0.00817	0.019926	0.006853	0.007952
96	0.007695	0.006264	-0.00761	-0.00629	-0.00206	0.012144	-0.02191	0.014121	0.002584	-0.04479	0.007708
97	0.051528	0.064925	0.139838	-0.00785	0.140272	-0.0017	0.094564	0.043809	0.014278	-0.0268	0.009957
98	-0.00277	-0.00636	-0.0044	0.000387	0.000245	0.003953	0.003381	-0.00925	0.015136	-0.01218	-0.01176
99	-0.01695	-0.01904	-0.01391	-0.00634	-0.0096	-0.01167	-0.00714	0.015327	0.037773	-0.04486	-0.04144
100	-0.00253	-0.03942	0.000287	-0.00162	-0.0104	-0.00791	0.015011	0.012705	0.000511	0.051558	-0.00776
101	0.038553	0.038934	0.020822	0.008824	0.02912	-0.00618	0.039818	0.012222	0.003004	0.055301	-0.01981
102	20.08256	22.0655	21.88912	25.51477	25.21964	24.62632	26.94317	31.86088	26.91411	27.25646	22.29999
103	225.4929	405.3448	526.8874	120.9771	325.2044	342.6023	238.1237	190.7135	318.1034	372.3564	388.5498
104	1.806983	11.33704	158.934	6.400592	148.5316	83.37258	42.69182	55.89904	41.28814	66.21563	107.6791
105	4940.836	9143.669	11701.28	3171.588	8380.184	8619.023	6667.848	6469.452	8757.255	10326.68	8979.293
106	6023.309	11369.32	10686.13	13422.32	8157.011	10225.97	13639.24	6193.168	11191.16	11179.28	10106.16
107	16.91858	50.75031	2840.868	368.0564	2693.752	1186.048	1814.125	1202.631	390.5166	1270.804	879.8373
108	831.5047	1683.925	1368.007	745.8935	1687.127	1458.946	1174.43	743.3351	1112.953	2915.779	1524.581
109	20.61374	17.40193	14.05581	25.49092	17.61654	18.8564	29.38773	31.06136	20.25168	28.60771	16.51617
110	44.30809	31.09926	79.34961	42.87241	59.5101	163.2736	16.60932	116.1217	70.78866	53.53439	62.09092
111	0.304381	0.399528	4.737576	1.753472	9.095175	30.71155	1.35122	37.55324	7.11303	6.561592	10.14637
112	889.6788	516.0841	1108.719	1089.205	1044.517	3076.836	458.6309	3602.726	1428.878	1523.692	1042.79
113	1534.474	966.2626	2626.943	4460.9	2108.601	5874.763	1415.768	4760.981	2767.218	1237.276	1810.991
114	1.077075	2.130296	60.07712	47.15983	137.0738	649.1224	18.86659	654.8741	71.2234	157.6164	110.5639
115	99.66185	22.9302	126.9306	190.7244	221.6811	653.0834	27.61394	449.3644	206.1707	326.4172	227.8467
