# Supplementary material for: Sheltering Behavior and Locomotor Activity in 11 Genetically Diverse Common Inbred Mouse Strains Using Home-Cage Monitoring
Source: PLoS One. 2014 Sep 29;9(9):e108563. doi: 10.1371/journal.pone.0108563 (PMC4180925; doi:10.1371/journal.pone.0108563)
Supplement: Table S4 — PC analysis varimax rotated solution matrix. 20 key parameters are indicated (bold). (PDF) [file pone.0108563.s004.pdf]

Rotated Component Matrix

	Component																								
	1	2	3	4	5	6	7	8	9	10	11	12	13	14	15	16	17	18	19	20	21	22	23	24	25	26
Short arrest duration - light	0.94																									
Short arrest number - light	0.93																									
Long movement number - light	0.93																									
Short movement number - light	0.91																									
Activity number - light	0.91																									
Long movement distance - light	0.90																									
Long arrest number - darklight index	-0.89																									
**Activity duration - light**	**0.89**																									
Activity number - darklight index	-0.89																									
Long arrest number - light	0.88																									
Short movement distance - light	0.86																									
Long arrest duration - darklight index	-0.85																									
Feeding zone duration - darklight index	-0.83																									
Spout zone duration - darklight index	-0.82																									
Long shelter visit duration - light	-0.80																									
Feeding zone duration - light	0.79																									
Spout zone duration - light	0.76																									
**Activity duration - darklight index**	**-0.74**																									
OnShelter zone number - light	0.68													0.48												
Long arrest duration - light	0.67									-0.45																
OnShelter zone duration - darklight index	-0.61											0.48														
Short shelter visit duration - light	0.51						0.49																			
Short shelter visit number - light	0.49	0.47																								
Long shelter visit number - light																										
Long movement number - dark		0.94																								
Short arrest number - dark		0.94																								
Long arrest number - dark		0.93																								
Long movement distance - dark		0.90																								
**Activity duration - dark**		**0.89**																								
Activity number - dark		0.89																								
Short movement number - dark		0.88																								
Short arrest duration - dark		0.85																								
Short movement distance - dark		0.80	0.50																							
Short shelter visit number - dark		0.77																								
**Long shelter visit fraction of total visits**		**-0.76**																								
Mean long arrest duration - dark		-0.73																								
**Activity change in response to to dark**		**0.66**																								
**Mean long arrest duration - light**		**-0.62**								**-0.53**																
**Long movement threshold**			**0.89**																							
Long movement fraction of total movement			-0.86																							
Mean short movement distance - dark			0.83																							
Mean short movement distance - light			0.81																							
Mean long movement distance - dark			0.81																							
Mean long movement distance - light			0.77																							
**Long movement max. velocity**			**0.62**																							
Long arrest number - habituation ratio light				0.88																						
Activity number - habituation ratio light				0.85																						
Long arrest duration - habituation ratio light				0.84																						
Feeding zone duration - habituation ratio light				0.80																						
Long shelter visit duration - habituation ratio light				-0.52																						
Spout zone duration - habituation ratio light				0.44																0.44						
Long shelter visit number - dark					-0.90																					
**Long shelter visit threshold**					**0.80**																					
Mean long shelter visit duration					0.79																					
Long shelter visit number - darklight index					-0.70																					
**Long shelter visit duration - dark**		**-0.50**			**-0.55**																					
Long shelter visit duration - darklight index	0.46	-0.43			-0.52																					
Mean activity duration - habituation ratio light						0.86																				
**Mean activity duration - light**						**0.77**																				
Mean activity duration - darklight index						-0.77																				
**Activity duration - habituation ratio light**				**0.60**		**0.64**																				
**Activity change in anticipation of dark**																										
Short shelter visit duration - dark							0.77																			
Mean short shelter visit duration - dark		-0.48					0.71																			
Mean short shelter visit duration - light							0.71																			
**Short shelter visit threshold**		**-0.48**					**0.70**																			
**Mean activity duration - dark**							**0.56**																			
Long arrest duration - habituation ratio dark								0.79																		
Feeding zone duration - habituation ratio dark								0.70																		
Mean long arrest duration - habituation ratio dark								0.67																		
Long arrest duration - dark	-0.40							0.59																		
Feeding zone duration - dark								0.52																		
Mean short arrest duration - dark		-0.43							0.77																	
Mean short arrest duration - light									0.75																	
**Long arrest threshold**		**-0.48**							**0.74**																	
Mean long arrest duration - darklight index										0.80																
Mean long arrest duration - habituation ratio light										-0.80																
Activity number - habituation ratio dark											0.75															
Long arrest number - habituation ratio dark											0.74															
**Activity duration - habituation ratio dark**											**0.64**		**0.58**													
Long shelter visit duration - habituation ratio dark											-0.60															
OnShelter zone change in response to dark												0.72														
OnShelter zone duration - habituation ratio dark												0.63														
OnShelter zone change in response to light												-0.59														
Mean short movement distance - habituation ratio dark													0.72													
Mean activity duration - habituation ratio dark													0.65													
Mean short arrest duration - habituation ratio dark													-0.64													
OnShelter zone duration - dark														0.67												
OnShelter zone duration - light	0.58													0.62												
**OnShelter zone number - dark**		**0.47**												**0.60**												
Mean short arrest duration - darklight index															0.69											
Mean short arrest duration - habituation ratio light															-0.54											
Spout zone change in anticipation dark																-0.78										
Spout zone change in response to dark																-0.68										
Feeding zone change in anticipation dark																0.62										0.49
Feeding zone change in response to dark																0.59										
Spout zone duration - habituation ratio dark																	0.80									
Spout zone duration - dark		0.51															0.59									
Mean short movement distance - habituation ratio light																		0.71								
Mean short movement distance - darklight index																		-0.53								
OnShelter zone duration - habituation ratio light				0.43														0.52								
Mean long movement distance - habituation ratio dark																			0.73							
Spout zone change in response to light																				0.80						
Spout zone change in anticipation light																				0.57						
Mean long movement distance - habituation ratio light																					-0.75					
Mean long movement distance - darklight index																					0.75					
**Activity change in anticipation of light**																						**0.74**				
**Activity change in response to to light**	**0.43**																					**0.49**				
Feeding zone change in response to light																										
Mean short shelter visit duration - habituation ratio light																							0.82			
Mean short shelter visit duration - darklight index																							-0.79			
OnShelter zone change in anticipation light																								0.73		
Feeding zone change in anticipation light																								-0.67		
Mean short shelter visit duration - habituation ratio dark																									0.75	
OnShelter zone change in anticipation dark																										-0.85
